# Chromosome‐level genome assembly of *Iodes seguinii* and its metabonomic implications for rheumatoid arthritis treatment

**DOI:** 10.1002/tpg2.20534

**Published:** 2024-11-27

**Authors:** Xun Gong, Hantao Zhang, Yinluo Guo, Shaoshuai Yu, Min Tang

**Affiliations:** ^1^ Department of Rheumatology & Immunology Affiliated Hospital of Jiangsu University Zhenjiang China; ^2^ School of Life Sciences Jiangsu University Zhenjiang China; ^3^ Department of Pharmacy Affiliated People's Hospital of Jiangsu University Zhenjiang China

## Abstract

*Iodes seguinii* is a woody vine known for its potential therapeutic applications in treating rheumatoid arthritis (RA) due to its rich bioactive components. Here, we achieved the first chromosome‐level assembly of the nuclear genome of *I. seguinii* using PacBio HiFi and chromatin conformation capture (Hi‐C) sequencing data. The initial assembly with PacBio data produced contigs with an N50 length of 9.71 Mb, and Hi‐C data anchored these contigs into 13 chromosomes, achieving a total length of 273.58 Mb, closely matching the estimated genome size. Quality assessments, including BUSCO, long terminal repeat assembly index, transcriptome mapping rates, and sequencing coverage, confirmed the high quality, completeness, and continuity of the assembly, identifying 115.28 Mb of repetitive sequences, 1062 RNA genes, and 25,270 protein‐coding genes. Additionally, we assembled and annotated the 150,599 bp chloroplast genome using Illumina sequencing data, containing 121 genes including key DNA barcodes, with maturase K (*matK*) proving effective for species identification. Phylogenetic analysis positioned *I. seguinii* at the base of the *Lamiales* clade, identifying significant gene family expansions and contractions, particularly related to secondary metabolite synthesis and DNA damage repair. Metabolite analysis identified 84 active components in *I. seguinii*, including the discovery of luteolin, with 119 targets predicted for RA treatment, including core targets like *AKT1*, toll‐like receptor 4 (*TLR4*), epidermal growth factor receptor (*EGFR*), tumor necrosis factor (*TNF*), *TP53*, *NFKB1*, janus kinase 2 (*JAK2*), *BCL2*, mitogen‐activated protein kinase 1 (*MAPK1*), and spleen‐associated tyrosine kinase (*SYK*). Key active components such as flavonoids and polyphenols with anti‐inflammatory activities were highlighted. The discovery of luteolin, in particular, underscores its potential therapeutic role. These findings provide a valuable genomic resource and a scientific basis for the development and application of *I. seguinii*, addressing the genomic gap in the genus *Iodes* and the order *Icacinales* and underscoring the need for further research in genomics, transcriptomics, and metabolomics to fully explore its potential.

## Core Ideas

- First chromosome‐level genome assembly of *Iodes seguinii* using PacBio HiFi and chromatin conformation capture, providing key resources.
- Identified 84 bioactive compounds in *I. seguinii*, including luteolin, with potential for arthritis treatment.
- Revealed 119 therapeutic targets, such as AKT1, toll‐like receptor 4, and tumor necrosis factor, showing potential for autoimmune disease treatment.
- Expanded gene families linked to metabolites offer insights into *I. seguinii*'s evolutionary adaptation.
- Network pharmacology and docking show luteolin and flavonoids bind to therapeutic targets, aiding drug development.

**Abbreviations**
AKT1: AKT serine/threonine kinase 1
BCL2: BCL2 apoptosis regulator
CHI: chalcone isomerase
CHS: chalcone synthase
EGFR: epidermal growth factor receptor
GO: gene ontology
Hi‐C: chromatin conformation capture
HOG: hierarchical orthologous group
JAK2: janus kinase 2
KEGG: Kyoto encyclopedia of genes and genomes
MAPK1: mitogen‐activated protein kinase 1
matK: maturase K
NF‐κB: nuclear factor kappa‐light‐chain‐enhancer of activated B cells
OPLS‐DA: orthogonal partial least squares discriminant analysis
PCA: principal component analysis
PCR: polymerase chain reaction
psbA‐trnH: intergenic spacer region
QC: quality control
RA: rheumatoid arthritis
rbcL: ribulose‐1,5‐bisphosphate carboxylase large subunit
SMILES: simplified molecular input line entry specification
SYK: spleen‐associated tyrosine kinase
TLR4: toll‐like receptor 4
TNF: tumor necrosis factor

## 1 INTRODUCTION

Globally, the family *Icacinaceae* comprises approximately 58 genera and 400 species, primarily distributed in tropical and subtropical regions, with the Southern Hemisphere serving as their predominant habitat (Allen et al., 2015). In China, 13 genera and 25 species of *Icacinaceae* are found, mainly in the southern and southwestern regions. Specifically, 10 genera have been identified in Yunnan Province: *Apodytes*, *Gomphandra*, *Gonocaryum*, *Natsiatum*, *Nothapodytes*, *Pittosporopsis*, *Platea*, *Mappianthus*, and *Iodes*. Among these, several genera include medicinal plants with notable pharmacological properties. For example, *Mappianthus iodoides*, from the genus *Mappianthus*, is a traditional medicinal plant used by the Dai people to treat irritability, frequent urination, and traumatic injuries, while the Yao people use its roots and stems to treat jaundice hepatitis, menstrual disorders, and snake bites (Hazarika et al., 2023). The genus *Iodes* is rich in active compounds and exhibits therapeutic effects in treating conditions such as rheumatism, nephritis, dysuria, and swelling pain by dispelling wind and cold, removing dampness, and promoting blood circulation (Gan et al., 2008; Ramesha et al., 2013).

The *Iodes* genus in China includes several species, such as *Iodes cirrhosa*, *Iodes balansae*, *Iodes vitiginea*, and *Iodes seguinii*, predominantly found in Yunnan Province. Research has unveiled multiple phenolic compounds in *I. cirrhosa*, like lignans, phenylpropanoids, and simple phenols, with potential applications in treating inflammation, rheumatic diseases, nephritis, and cancer (Gan et al., 2008; Murray & Young, 2019; Ramesha et al., 2013; Thi Ngoc et al., 2022). This species, however, is the sole member of the *Iodes* genus subjected to genomic analysis, revealing a chloroplast genome of 151,994 base pairs, housing 80 protein‐coding genes, 28 tRNA genes, and four rRNA genes (L. Wang et al., 2019). Therefore, the lack of fundamental genomic research has significantly hindered the study of the *Iodes* genus.

**FIGURE 1:**
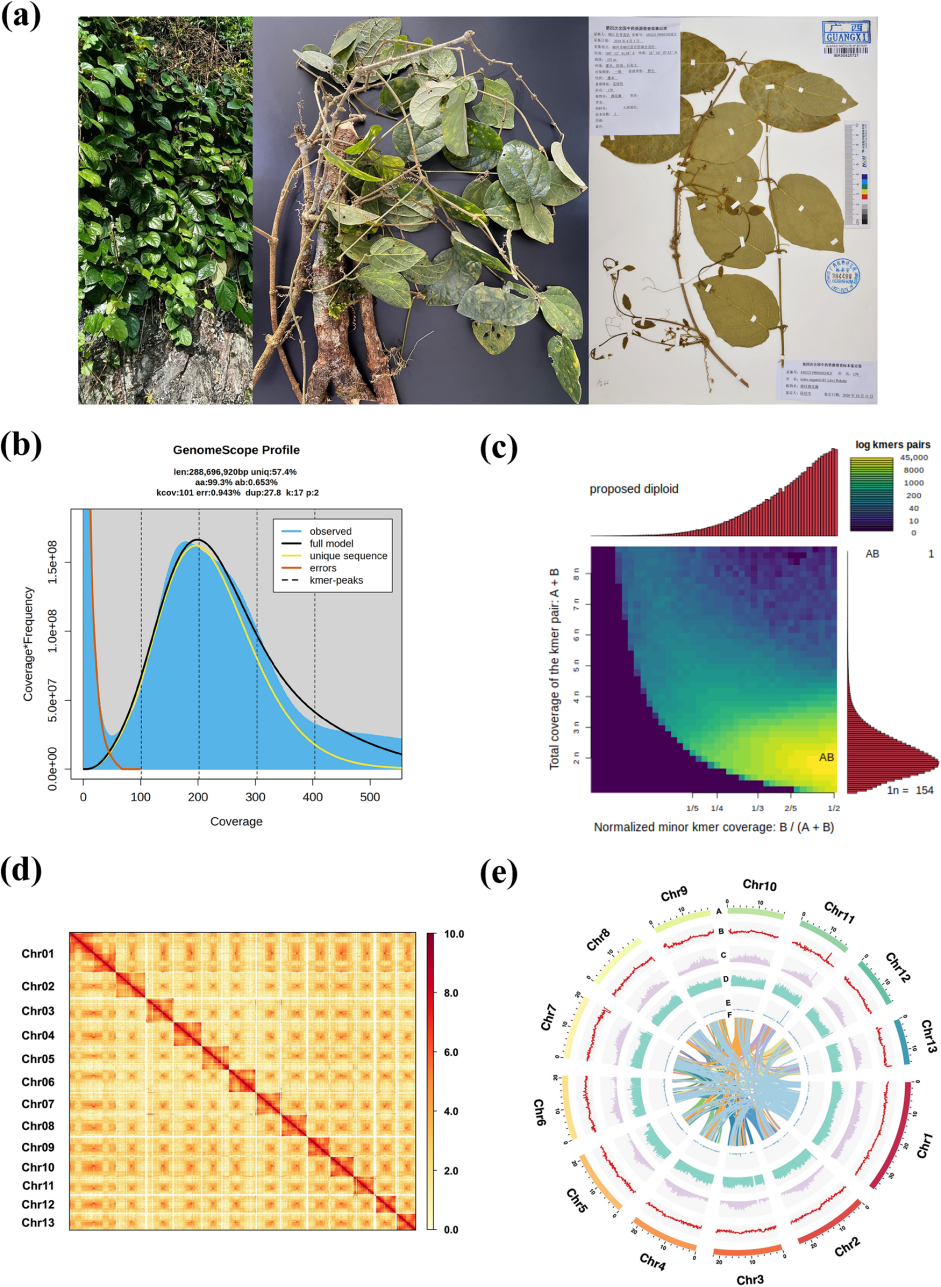
Comprehensive de novo genome assembly analysis. (a) Morphological characteristics of *I*. *seguinii*. (b) GenomeScope projection of genome size and heterogeneity using a 17‐mer analysis. (c) Smudgeplot estimation depicting genome ploidy. (d) 3D‐DNA produced Hi‐C interaction map showcasing chromosomal interactions. (e) Tracks A–F represent the distribution of chromosome karyotypes, guanine–cytosine (GC) content density, gene density, density of repeat sequences, RNA gene density, and genomic collinearity, respectively, with each density measured over 100‐kb genomic windows.

*Iodes seguinii* is distinguished by its nodular lenticels and is prized for its sweet, pungent fruit, as well as its medicinal properties (Figure 1a). In Guangxi, this plant is particularly valued for treating nephritis and is sometimes used as a substitute for *Stephania tetrandra*, a traditional remedy known for alleviating rheumatic arthralgia and edema (Jiang et al., 2020). The therapeutic attributes of *I. seguinii* suggest that it may be beneficial in managing rheumatic immune disorders. Despite its potential, research on the medicinal molecules and mechanisms of action of *I. seguinii* is almost nonexistent, highlighting a significant gap in the understanding of this plant's pharmacological potential.

In this study, we have documented high‐quality nuclear and chloroplast genomes of *I. seguinii*. Moreover, we have investigated the potential therapeutic applications of active compounds found in the roots, stems, and leaves of this plant for the treatment of rheumatoid arthritis (RA). These findings provide a robust molecular genetic basis for further genetic enhancement, quality improvement, and biosynthetic studies of *I. seguinii*.

## 2 MATERIALS AND METHODS

### 2.1 Sample collection, library construction, and sequencing

Entire plants of *I. seguinii* growing naturally were collected from Xichou County in the Wenshan Zhuang and Miao Autonomous Prefecture, Yunnan Province. Each plant was comprehensively sampled, including root, stem, and leaf tissues, and individually labeled as Xichou#1, Xichou#2, and Xichou#3. The samples were quickly frozen using liquid nitrogen and subsequently stored at −80°C. Observations and photographic records were made to document the size, shape, color, surface characteristics, and texture of the fresh plants.

For nuclear genome assembly from Xichou#1, genomic DNA was extracted from young leaves using the UNlQ‐10 Column Trizol Total RNA Isolation Kit and assessed via agarose gel electrophoresis for quality. Single molecular real‐time sequencing libraries were prepared following Pacific Biosciences' standard protocols. The genomic DNA was sheared to approximately 20 kb, and damaged ends were repaired and linked with blunt‐end adaptors. The libraries were then sequenced on the PacBio Sequel II platform. The workflow for constructing Illumina libraries is as follows: genomic DNA is fragmented into target fragments of approximately 350 bp using ultrasonic shearing. The fragments undergo end repair, addition of an A base, adapter ligation, target fragment selection, and polymerase chain reaction (PCR) amplification to construct the short fragment sequencing library. For the chromatin conformation capture (Hi‐C) library construction, leaf samples were first fixed with formaldehyde, and chromatin was extracted. This chromatin was then digested using 400 U of DPNII enzyme at 37°C. The DNA ends were biotin‐labeled and ligated with T4 DNA ligase from NEB. Post ligation, proteinase K was used for reverse cross‐linking. The DNA fragments were purified and dissolved, then fragmented to 350–500 bp, and biotin‐labeled fragments were isolated with Dynabeads MyOne Streptavidin C1. The Illumina libraries and Hi‐C libraries were both sequenced using the Illumina NovaSeq 6000 sequencing platform.

**Core Ideas**

- First chromosome‐level genome assembly of *Iodes seguinii* using PacBio HiFi and chromatin conformation capture, providing key resources.
- Identified 84 bioactive compounds in *I. seguinii*, including luteolin, with potential for arthritis treatment.
- Revealed 119 therapeutic targets, such as AKT1, toll‐like receptor 4, and tumor necrosis factor, showing potential for autoimmune disease treatment.
- Expanded gene families linked to metabolites offer insights into *I. seguinii*'s evolutionary adaptation.
- Network pharmacology and docking show luteolin and flavonoids bind to therapeutic targets, aiding drug development.

For chloroplast genome assembly, total RNA was extracted from leaf samples using the RNAprep Pure Plant Plus Kit. Equal amounts of total RNA from different samples were mixed, and this mixture was then used for library construction and sequencing. The Illumina library construction process involves using magnetic beads with Oligo (dT) to enrich eukaryotic mRNA, adding Fragmentation Buffer to randomly break the mRNA, using mRNA fragments as templates to synthesize the first strand of cDNA with six‐base random primers, and then adding buffer, dNTPs, RNase H, and DNA polymerase I for second‐strand cDNA synthesis. The double‐stranded cDNA is purified using AMPure XP magnetic beads, followed by end repair, A‐tailing, and sequencing adapter ligation, with further size selection using AMPure XP beads, and finally enriched by PCR to complete the cDNA library construction. High‐throughput sequencing of both ONT and Illumina libraries was performed on PromethION P48 and Illumina HiSeq X Ten platforms, respectively.

### 2.2 Methodology for species identification using DNA barcoding

For precise species identification, the ribulose‐1,5‐bisphosphate carboxylase large subunit (*rbcL*), maturase K (*matK*), and intergenic spacer region (*psbA‐trnH*) sequences were amplified using genomic DNA (Figure S1). Primer sequences utilized for amplifying these DNA barcodes, along with the specific PCR reaction protocols, are detailed in Table S1. The sequencing results of *rbcL* and *matK* were compared with sequences in the BOLD Systems database (https://www.boldsystems.org), while the *psbA‐trnH* sequences were aligned against the Nucleotide Sequence Database (NT). Species identification was based on comparison scores, sequence similarity, and *E*‐value.

### 2.3 Genome survey, assembly, and quality assessment

As illustrated by the bioinformatics pipeline in Figure S2, Jellyfish (v2.2.10) calculates k‐mer frequencies from genomic Illumina sequencing data. Subsequently, a histogram of these frequencies is generated using the same software. GenomeScope (v2.0) then evaluates the nuclear genome's total length, heterozygosity, and repeat content. Finally, Smudgeplot (v0.2.5) determines the k‐mer coverage range for nuclear genome ploidy assessment and generates smudge plots. The parameters set for this assessment are default.

Hifiasm software (v0.19.4‐r575) leverages quality‐controlled genomic PacBio HiFi and Hi‐C sequencing data to construct accurate and contiguous haploid genome assemblies, further processed into FASTA format using gfatools (v0.4‐r214‐dirty). HiCUP software (v0.9.2) aligns Hi‐C sequencing data to these contigs, which are assembled into scaffolds using YaHS software (v1.2a.1), guided by the recognition site of HindIII. The assembly is refined and validated using Juicer software (v1.1) and Juicebox software (v1.11.08), which utilize hic and assembly files to correct assembly errors and finalize chromosome‐level structures. BUSCO software (v5.4.6) evaluates the completeness and accuracy of the assembly by comparing it to the Embryophyta_odb10 dataset, a comprehensive plant orthologous gene database. Alignment and coverage analyses are performed using HISAT2 (v2.2.1), minimap2 (v2.17‐r941), and samtools (v1.18), supplemented by mosdepth (v0.3.6) for precise coverage calculations, ensuring a robust assessment of the sequencing and assembly processes.

### 2.4 Genome annotation

To identify long terminal repeats (LTRs) in the nuclear genome, LTRharvest (v1.6.1) and LTR_FINDER_parallel (v1.2) are employed (Ellinghaus et al., 2008; Ou & Jiang, 2019). Results from both software are integrated, and the long terminal repeat assembly index (LAI) score is calculated using LTR_retriever (v2.9.0) (Ou & Jiang, 2018). RepeatModeler (v2.0.4) predicts repeat sequences de novo in the nuclear genome (Abrusan et al., 2009). Predicted results are merged with repeat sequences from the Repbase and Dfam databases and annotated using RepeatMasker (v4.1.4) (Jurka et al., 2005; Storer et al., 2021; Tarailo‐Graovac & Chen, 2009). After that, tRNAscan‐SE (v2.0.11) and RNAmmer (v1.2) are used to predict tRNA and rRNA genes, respectively (Chan et al., 2021; Lagesen et al., 2007). The INFERNAL software's cmscan tool (v1.1.4) compares covariance models from the Rfam database with the nuclear genome to identify RNA genes (Kalvari et al., 2021; Nawrocki & Eddy, 2013).

Three strategies are utilized for annotating protein‐coding genes in the nuclear genome: de novo prediction, homology‐based prediction, and transcriptome‐based prediction. PASA (v2.5.2) aligns transcriptome splicing results with the nuclear genome (Haas, 2003). The correct gene structures from PASA are used for training AUGUSTUS (v3.5.0) and GlimmerHMM (v3.0.4), while GeneMark‐ET (v4.71_lic) is trained with intron positions from the nuclear genome (Bruna et al., 2020; Majoros et al., 2004; Stanke et al., 2008). Genome sequences and annotations from the NCBI Genome database for *Coffea arabica* (*C. arabica*) and *Coffea eugenioides* (*C. eugenioides*), along with transcriptome alignment results, are input into GeMoMa (v1.9) for homology‐base d prediction (Keilwagen et al., 2018). Protein sequences from the Lamiaceae family in the UniProt database are used for GenomeThreader (v1.7.1) homology prediction. EVidenceModeler (v1.1.1) integrates results from de novo, protein, and transcript‐based homology predictions to form the final gene structure (Haas et al., 2008).

Predicted protein‐coding genes are functionally annotated using similarity comparison methods. DIAMOND (v2.0.15.153) searches for homologous genes in the UniProt and NR databases with parameters set to evaluate 1e‐5 and ‐k 1 (Buchfink et al., 2021; UniProt, 2023). The eggNOG‐mapper and KAAS websites provide gene ontology (GO) and Kyoto encyclopedia of genes and genomes (KEGG) annotations for these genes (Cantalapiedra et al., 2021; Moriya et al., 2007). Finally, InterproScan (v5.61‐93.0) performs structural domain annotations using databases like Pfam and also provides GO terms and InterPro domain annotations (Jones et al., 2014).

### 2.5 Homologous gene clustering and phylogenetic tree construction

Complete genome sequences, genome annotation files, coding sequences (CDS), and protein sequences for nine Lamiaceae plants were downloaded from the NCBI Genome database: *Anisodus acutangulus*, *Arabidopsis thaliana*, *Capsicum annuum*, *C. eugenioides*, *Lycium barbarum*, *Solanum dulcamara*, *Sesamum indicum*, *Solanum lycopersicum*, and *Salvia miltiorrhiza*. Based on the genome annotations, genes and their longest transcripts were identified for subsequent analysis. OrthoFinder (v2.5.5) was used for orthologous and paralogous gene clustering. The clustered single‐copy orthologous genes were inputted into MAFFT (v7.520) for multiple sequence alignment. Protein alignments were converted to corresponding CDS alignments using PAL2NAL (v14), and conserved sites were extracted from these alignments with trimAl (v1.4. rev15). These conserved sites were then used as input to create a supermatrix with the phylotools package (v0.2.2), which served as the basis for constructing a phylogenetic tree using IQ‐TREE (v2.2.5).

### 2.6 Divergence time estimation

Based on the results of the CDS multiple sequence alignment, phylogenetic tree, and species divergence times obtained from the TimeTree website (https://timetree.org), the mcmctree program in PAML (v4.10.6) was used to calculate species divergence times.

### 2.7 Gene family expansion and contraction analysis

Based on the results of homologous gene clustering, phylogenetic tree construction, and species divergence time estimation, CAFÉ (v5.0.0) was used to analyze changes in gene family size throughout evolutionary history. Using the nuclear genome's protein‐coding genes as a background gene set, significant expansions or contractions were analyzed for GO and KEGG enrichment using the clusterProfiler package (v4.6.2).

Based on the results of CDS multiple sequence alignment and phylogenetic tree construction, the codeml program in PAML software was used to detect Darwinian positive selection driving protein evolution. This study utilized the branch of *A. acutangulus* in the phylogenetic tree as the foreground branch, with other branches serving as background lineages. The branch‐site model was used to calculate selective pressure on the foreground branch and to identify genes under positive selection through likelihood tests (*p*‐value ≤ 0.05).

### 2.8 Genome polyploidization analysis

Using BlastP software (v2.14.0), protein sequence alignments of *A. acutangulus* were performed and compared with the protein sequences of *L. barbarum*, *S. dulcamara*, *S. indicum*, and *S. miltiorrhiza* (Camacho et al., 2009). Based on the alignment results, MCScanX software was used to detect collinear blocks within the nuclear genome of *A. acutangulus*, and the results were visualized on the SynVisio website (https://synvisio.github.io/) (Y. Wang et al., 2012). WGDI software (v0.6.5) was employed to analyze collinear blocks within and between species and to calculate the substitution per synonymous site (Ks), plotting the results as frequency distribution charts (Sun et al., 2022). JCVI software (v1.3.8) was used to identify collinear blocks between species and to generate macro‐synteny plots (H. Tang et al., 2008). The parameters used for running these three software applications were set to default.

### 2.9 Gene identification and evolutionary analysis

The protein domain HMM configuration files for chalcone synthase (CHS) and isomerase were downloaded from the Pfam database (http://pfam.xfam.org). HMMER software (v2.3.2) and its subprogram hmmsearch were used to search for proteins with the same domains in the protein sequences of *A. acutangulus* (Finn et al., 2015). The search results were combined with gene functional annotations from eggNOG‐mapper to identify the CHS and chalcone isomerase (CHI) genes in *A. acutangulus*. Then, OMA standalone software (v2.6.0) was used for hierarchical orthologous clustering based on the protein sequences of 10 plants, and the clustering results that included the CHS and CHI genes were visualized using the pyHam package (v1.1.12) (Altenhoff et al., 2019; Train et al., 2019). The parameters set for gene identification, clustering, and visualization of clustering results were default.

To explore the gain or loss of genes encoding these two key rate‐limiting enzymes during adaptive evolution, this study used the protein sequences of *I. seguinii*, *A. thaliana*, and the aforementioned eight Lamiaceae species as input files. The OMA standalone software was employed to identify hierarchical orthologous groups (HOGs).

### 2.10 De novo assembly and annotation of the chloroplast genome

To better understand the evolutionary relationships and genetic diversity among plant species, sequencing and assembling the chloroplast genome provides crucial insights into conserved genetic markers. This approach provides a foundation for comparing genetic similarities and differences that can shed light on plant adaptation and speciation. As illustrated by the bioinformatics pipeline in Figure S3, the whole genome Illumina sequencing data, after quality assessment and control, were assembled into the chloroplast genome using GetOrganelle (v1.7.7.0) (Jin et al., 2020). After assembly, the results were viewed in Bandage (v0.8.1) to confirm circular assembly (Wick et al., 2015). Subsequently, the chloroplast genome sequences of two configurations were aligned with those of *I. cirrhosa*, *C. arabica*, *Solanum tuberosum*, *S. lycopersicum*, *C. annuum*, *Nicotiana tabacum*, and *A. thaliana* using MAFFT (v7.520) (Rozewicki et al., 2019). Conserved sites were extracted using trimAl (v1.4.rev15) (Capella‐Gutierrez et al., 2009), and the results were used to construct a phylogenetic tree with IQ‐TREE (v2.2.5) (Minh et al., 2020).

The chloroplast genome annotation file of *I. cirrhosa* was downloaded from the NCBI Nucleotide database (https://www.ncbi.nlm.nih.gov/nuccore) and used as a reference for annotating the chloroplast genome of *A. acutangulus* on the CPGAVAS2 website (http://47.96.249.172:16019/analyzer/home) (Shi et al., 2019). The GenBank file was then submitted to the CPGView website (http://47.96.249.172:16085/cpgview/home) to visualize the annotation results as a feature map (S. Liu et al., 2023). The annotation results from CPGAVAS2 and the visualization results from CPGView were combined to analyze the chloroplast genome's length, lengths of various regions (large single copy [LSC], small single copy [SSC], and inverted repeat [IR]), number of protein‐coding genes, and number of noncoding genes.

### 2.11 Sample preparation, extraction for metabolite detection

Rhizome and leaf samples were flash‐frozen and ground into a fine powder, which was then mixed with a methanol/acetonitrile/water solution for centrifugation. The resulting supernatant was dried and subsequently redissolved for analysis. High‐performance liquid chromatography‐tandem mass spectrometry (HPLC‐MS/MS) was employed to separate and analyze the samples. Quality control measures were incorporated throughout the process to ensure the reliability of the data. Metabolites were qualitatively and quantitatively analyzed by converting mass spectrometry data into mzXML format, followed by peak alignment, retention time correction, and peak area extraction. Identification of metabolites was performed against a laboratory‐built database. The detailed flowchart for the analysis of metabolite detection data was illustrated in Figure S4.

### 2.12 Metabolite detection results statistics and inter‐group difference analysis

Metabolites identified in both positive and negative ion modes were categorized based on their chemical classifications. The principal component analysis (PCA) was conducted using the ropls package (v1.34.0) to evaluate the reliability of metabolite analysis and the distribution trends between groups. Differences in metabolite abundance among roots, stems, and leaves were analyzed using *T*‐tests, fold change analysis, and orthogonal partial least squares discriminant analysis (OPLS‐DA) to identify significant differential metabolites (DMs). These DMs were further analyzed for expression patterns in root, stem, and leaf samples using the Mfuzz package (v2.26.0).

The relative abundances of differential metabolites were visualized with hierarchical clustering heatmaps using the pheatmap package (v1.0.12). Metabolite pathway annotations and enrichments were performed using the KEGG databases, and subsequent pathway enrichment analysis of the differential metabolites was conducted using the clusterProfiler package (v4.10.0).

### 2.13 Network pharmacology analysis and docking analysis

The physicochemical and pharmacokinetic properties of the detected metabolites were assessed using SwissADME. Simplified molecular input line entry specification (SMILES) codes were subsequently entered into SwissTargetPrediction for target identification. RA treatment targets were investigated using OMIM, GeneCards, and DisGeNET databases. Significant targets identified were analyzed using the VennDiagram package (v1.7.3), and enrichment analysis was performed with the clusterProfiler package. These targets were integrated into a network constructed in Cytoscape (v3.10.1), employing the NetworkAnalyzer plugin to identify key active components. Additionally, a protein–protein interaction (PPI) network was constructed using the STRING database and analyzed in Cytoscape with the CytoNCA plugin to determine essential therapeutic targets.

Key therapeutic target structures were retrieved from the RCSB protein data bank database. These structures were prepared in PyMOL (v2.5.5) by removing water and ligand molecules and subsequently processed in AutoDock Tools (v1.5.7) for docking simulations. Structures of active components were downloaded from PubChem, converted to mol2 format using Open Babel (v3.1.0), and further prepared in AutoDock Tools. Docking simulations between these key targets and active components were conducted using AutoDock Vina (v1.1.2) to evaluate their interaction efficacy.

## 3 RESULTS

### 3.1 Species identification

In addition to morphological observations, DNA barcoding was employed to identify the three plant specimens collected from Yunnan, China. By analyzing the *rbcL* gene, genus‐level identification was achieved, confirming that the specimens belong to the genus *Iodes*. However, the use of the *matK* gene provided precise species‐level identification, consistently confirming the target species (Table S2). Due to the limited number of *Iodes* sequences in the NT database, the *psbA‐trnH* sequence aligned with the chloroplast genome of *I. cirrhosa* (Table S3).

### 3.2 Genome assembly and completeness evaluation

An individual of *I. seguinii*, numbered as Xichou#1, was sequenced. The estimated genome size of *I. seguinii* was approximately 288.70 Mb (Figure 1b), with a diploid karyotype as determined by a k‐mer survey using Illumina short reads (Figure 1c). The heterozygosity level was estimated at 0.65%, and repetitive content accounted for 42.1% of the genome. A de novo assembly from 29 Gb (∼100× coverage) of HiFi reads, with an average read length of 18.52 kb, produced an assembled sequence of 273.58 Mb, comprising 355 contigs with an N50 size of 9.71 Mb (Table 1; Figure S5a). Utilizing 107 Gb of Hi‐C data, the sequence of *I. seguinii* was further organized by anchoring the contigs into 399 scaffolds, achieving an N50 size of 18.97 Mb. After manual correction, the sequence was ultimately assembled to a total size of 273.58 Mb, organized into 13 pseudochromosomes (Figure 1d). These pseudochromosomes range in size from approximately 14.92 Mb to 36.59 Mb (Table S4). Chromosome 1 is the longest and is composed of longer contig sequences, which is also observed in chromosomes 6 and 10. In contrast, the shortest chromosome, chromosome 13, contains shorter contig sequences, similar to chromosomes 2, 3, 4, 5, 7, 8, 9, 11, and 12 (Figure S5b).

**TABLE 1:** Quality assessment of the nuclear genome assembly of *I. seguinii*.

Items	Data
Plant material	Xichou#1 from Yunnan Province, China
Estimated genome size (Mb)	288.70
Estimated heterozygosity (%)	0.65
Sequencing platform (genome coverage)	
PacBio Sequel II (Gb)	28.97 (100×)
Illumina NovaSeq 6000 (DNA‐seq, Gb)	29.04 (100×)
Illumina NovaSeq 6000 (Hi‐C, Gb)	107.3 (371×)
ONT PromethION P48 (Gb)	51.82
Illumina HiSeq X (RNA‐seq, Gb)	24.77
Assembly statistics	
Total number of scaffolds	399.00
Scaffold N50 length (Mb)	18.97
Total number of contigs	355.00
Contig N50 length (Mb)	9.71
Assembly size (Mb)	273.58
GC content (%)	32.73
Repetitive content (%)	42.10
Assessment	
Genome BUSCOs (%)	97.40
LTR assembly index	11.44
Illumina mapping rate	87.64
ONT mapping rate	89.62

Abbreviations: GC, guanine–cytosine; LTR, long terminal repeat.

A comprehensive evaluation of the assembly quality was conducted. The Hi‐C interaction heatmap indicates that the contigs anchored to the 13 chromosomes are accurate in clustering, sorting, and orientation. The alignment rates of the transcriptome Illumina sequencing data and ONT sequencing data to the 13 chromosomes are 87.64% and 89.62% of their respective total data. Additionally, the assembly completeness of these 13 chromosomes is 97.4%, the HiFi sequencing coverage is 52.08×, and the LAI is 11.44, which falls under the “Reference” level. These results indicate that the chromosome‐level nuclear genome assembled in this study meets the cutting‐edge standards in terms of precision, completeness, and coherence.

### 3.3 Genome annotation

Transposons constitute the largest proportion of repetitive sequences at 22.32%. DNA transposons and retrotransposons, including SINE, LINE, and LTR, account for 4.17%, 0.17%, 3.89%, and 14.09% of the nuclear genome, respectively (Table S5). Microsatellites (simple repeats) are the most numerous, comprising 33.18% of the total repetitive sequences. Satellites, another type of tandem repeat, account for only 0.000511%. Chromosome 1 has the highest number of DNA transposons, LINEs, microsatellites, and SINEs. Chromosome 2 has the highest number of LTRs and satellites, while chromosome 5 has the highest content of repetitive sequences (Figure S6).

A total of 25,270 genes were predicted in the *I. seguinii* genome assembly using homology, transcript‐based, and ab initio gene prediction approaches, after excluding 115.28 Mb (42.14%) of repetitive sequences (Figure 1e; Table S6). The completeness of the genome annotation was assessed using BUSCO, revealing that the annotated gene set covered 1513 (93.74%) of the 1614 universal single‐copy genes found in the Embryophyta lineage (Table S7). Sequence similarity comparison methods were used to identify known functional genes homologous to the 25,270 protein‐coding genes in five major databases (NR, Swiss‐Prot, eggNOG, KEGG, InterPro, Pfam) and subsequently annotated the functions of 23,998 (94.97%) genes based on homology. Functional annotations for these genes were derived from at least one of the aforementioned databases. Additionally, 16,992 genes (67.24%) were associated with at least one GO annotation, and 9777 genes (38.69%) were associated with at least one KEGG pathway annotation (Table S8).

### 3.4 Orthologue identification and phylogenetic inference

**FIGURE 2:**
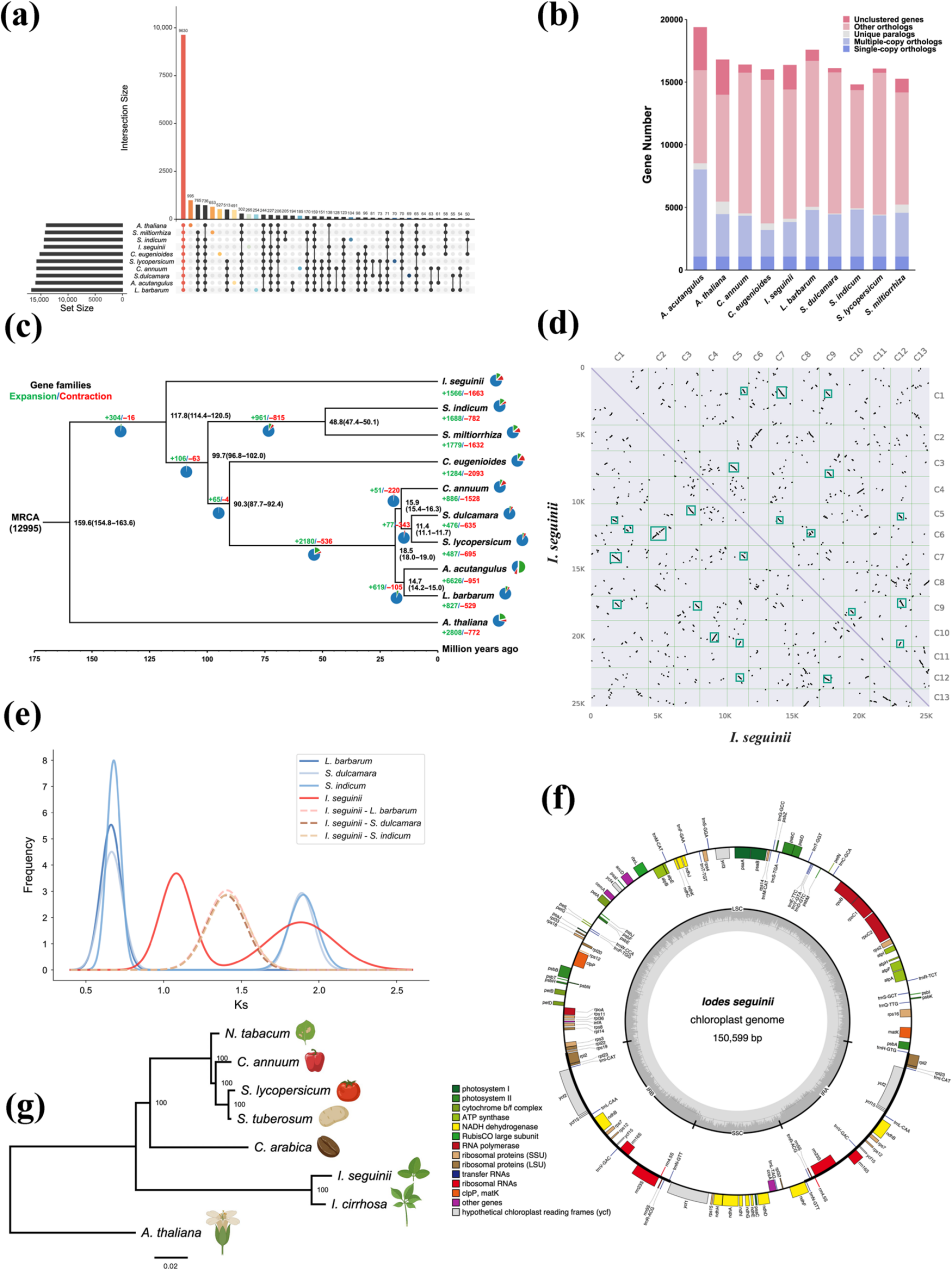
Gene family dynamics across species and phylogenetic inference. (a) Gene clustering analysis involving 10 different species. (b) Comprehensive overview of orthologous and paralogous gene relationships. (c) Phylogenetic tree illustrates the dynamic evolution of gene families among 10 species, with blue and red numbers on each branch indicating the number of gene families that have expanded and contracted, respectively. Pie charts adjacent to each branch detail the proportions of these expanded (blue) and contracted (red) gene families, while black numbers denote divergence times. (d) Self‐self synteny analysis of pseudo‐assembled genome. Depicting numerous inversions and duplications on a graph. (e) Ks distribution among *I. seguinii* and three other species, highlighting the synonymous substitution rates and revealing evolutionary events and divergence times. (f) Characterization of the chloroplast genome. (g) Phylogenetic tree inferred from the chloroplast genomes of eight plant species. ATP, adenosine triphosphate; LSU, large subunit; MRCA, most recent common ancestor; NADH, nicotinamide adenine dinucleotide (reduced form); SSU, small subunit.

The results from OrthoFinder indicated that the protein‐coding genes from *A. acutangulus*, *A. thaliana*, *C. annuum*, *C. eugenioides*, *L. barbarum*, *S. dulcamara*, *S. indicum*, *S. lycopersicum*, *S. miltiorrhiza*, and *I. seguinii* constitute 9630 gene families, including 1076 single‐copy orthologous genes (Figure 2a). Among the 25,270 protein‐coding genes in *I. seguinii*, 23,308 were clustered into 14,410 gene families, while the remaining 1962 genes did not belong to any gene family (Figure 2b). To determine the phylogenetic position of *I. seguinii* within the Lamiids, a maximum likelihood phylogenetic tree was constructed, with *A. thaliana* serving as the outgroup. The resulting rooted tree reveals that *I. seguinii* does not share a close evolutionary relationship with eight other Lamiids plants included in the study. Instead, the data suggest that *I. seguinii* may be situated at the base of the Lamiids evolutionary tree, indicating a more distant ancestral lineage within this clade (Figure 2c).

To date evolutionary events, a time‐calibrated phylogenetic tree was reconstructed using fossil calibration times. The analysis revealed that *A. acutangulus* and *L. barbarum*, two common medicinal herbs, diverged approximately 14.7 million years ago. Plus, *S. dulcamara* and *S. lycopersicum* diverged around 11.4 million years ago, and their common ancestor with *C. annuum* split around 15.9 million years ago. The divergence between these five Solanaceae plants and Rubiaceae plants (*C. eugenioides*) occurred approximately 90.3 million years ago. This lineage was sister to another *Lamiales* branch, which includes *S. indicum* (*Pedaliaceae*) and *S. miltiorrhiza* (*Lamiaceae*), diverging about 99.7 million years ago. *Sesamum indicum* and *S. miltiorrhiza* themselves diverged between 47.4 and 50.1 million years ago. The divergence of these eight plants from *I. seguinii* occurred between 114.4 and 120.5 million years ago. Serving as the outgroup, *A. thaliana* diverged from these nine Lamiales plants around 15.96 million years ago.

### 3.5 Expansion, contraction, and positive selection of gene families

In the 14,410 gene families of *I. seguinii*, 1566 families expanded and 1663 contracted. Genes in significantly expanded families (SEGs) were primarily involved in the synthesis of secondary metabolites such as ethylene, coumarins, monoterpenes, phenylpropanoids, flavonoids, and steroid hormones (Figure S7a,b). Conversely, genes in significantly contracted families (SCGs) were related to the synthesis of sesquiterpenes, phenylpropanoids, monoterpenes, glucosinolates, isoflavones, and zeatin, as well as the degradation of terpenoids and isoprenoids, and the metabolism of alkaloids, linoleic acid, triterpenes, and galactose (Figure S7c,d). Additionally, SEGs were enriched in the isoflavonoid biosynthesis pathway, while SCGs were enriched in GO terms for defense response to damage and herbivores. These enrichment analysis results provide insights into the adaptation of *I. seguinii* to its environment and the associated metabolic processes over its long‐term evolution. In the lineage containing *I. seguinii*, a total of 156 genes are under positive selection (*p*‐value < 0.05). These genes are primarily associated with DNA damage response mechanisms, including DNA recombinational repair (GO:0000725, ko03400), interstrand cross‐link repair (GO:0036297), double‐strand break repair (GO:0006302), and homologous recombination repair (GO:0000724, ko03440) (Figure S7e,f).

### 3.6 Genome collinearity and polyploidy events

The collinearity analysis of *I. seguinii* chromosomes identified 6646 collinear genes and 342 collinear blocks within the nuclear genome (Figure 2d; Figure S8). Subsequently, three medicinal plants were selected with *I. seguinii* to calculate Ks values based on the nuclear genome structure annotation, CDS sequences, and protein sequences (Figure 2e). Ks calculations for *S. dulcamara* and *S. indicum* indicated that they, along with *I. seguinii*, underwent an ancient polyploidy event (Ks ≈ 1.89) followed by divergence (Ks ≈ 1.40), consistent with the phylogenetic tree. Post divergence, *I. seguinii* experienced a separate polyploidy event (Ks ≈ 1.08), while *S. dulcamara* and *S. indicum* shared another event with *L. barbarum* (Ks ≈ 0.69). Divergence among these species occurred approximately 114.4–120.5 million years ago, suggesting a synonymous nucleotide substitution rate of approximately 5.81 × 10⁻⁹ to 6.12 × 10⁻⁹. Thus, the two polyploidy events in *I. seguinii* likely occurred 88.2–92.9 million years ago and 154.4–162.7 million years ago. Collinearity analysis revealed that each chromosome of *I. seguinii* had collinear blocks with multiple chromosomes of *S. dulcamara* and *S. indicum*, indicating no one‐to‐one collinearity relationship, likely due to the unique polyploidy events in these species (Figures S9 and S10).

### 3.7 Chloroplast genome assembly and annotation

The assembly results of the chloroplast genome of *I. seguinii* indicate a single isoform configuration with a total size of 150,599 bp. The chloroplast genome is composed of four regions: an LSC region of 84,147 bp, an SSC region of 18,932 bp, and two inverted repeats (IRs), A and B, each measuring 23,760 bp. (Figure 2f). It contains 121 genes, including 84 protein‐coding genes, 29 tRNA genes, and eight rRNA genes. Six protein‐coding genes (*ndhB*, *rpl2*, *rpl23*, *rps7*, *ycf15*, *ycf2*), six tRNA genes (*trnI‐CAT*, *trnL‐CAA*, *trnM‐CAT*, *trnN‐GTT*, *trnR‐ACG*, *trnV‐GAC*), and four rRNA genes (*rrn4.5S*, *rrn5S*, *rrn16S*, *rrn23S*) are duplicated; eight protein‐coding genes (*atpF*, *ndhA*, *ndhB*, *petB*, *petD*, *rpl2*, *rpoC1*, *rps16*) contain one intron each; and each of two protein‐coding genes (*clpP* and *ycf3*) contain two introns (Table S9). Subsequently, the chloroplast genome sequence was compared to those of *A. thaliana* and six related species to construct a phylogenetic tree (Figure 2g). The analysis revealed that *I. cirrhosa* and *I. seguinii* belong to the same sister clade within the genus *Iodes*.

### 3.8 Identification and analysis of differential metabolites

**FIGURE 3:**
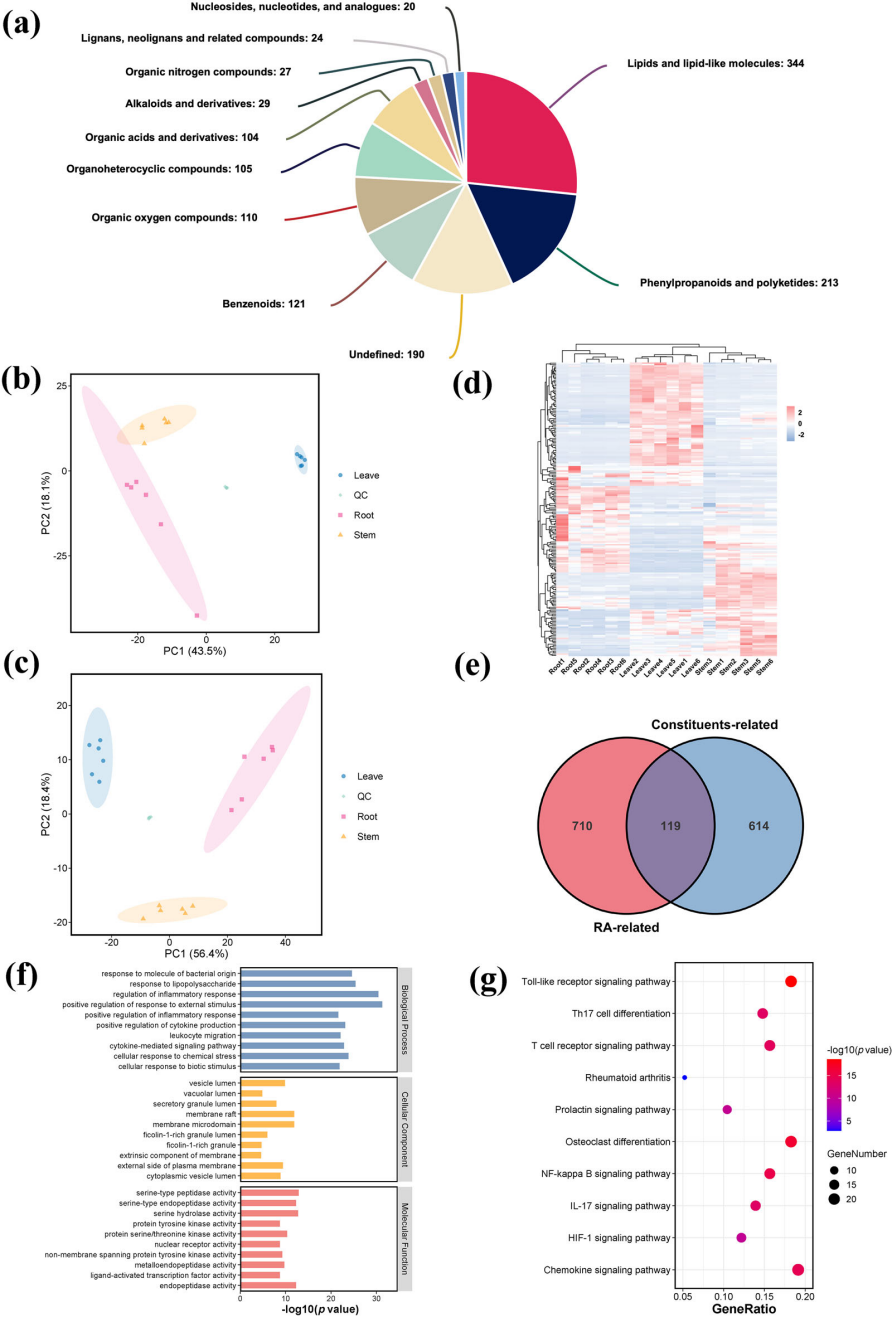
Detailed metabolomic profiling and target analysis. (a) Classification of metabolites identified in roots, stems, and leaves, displaying various compound categories. The principal component analysis (PCA) of root, stem, leaf, and quality control (QC) samples in (b) positive and (c) negative ion mode showed clear separation of tissue types. (d) Hierarchical clustering analysis of differential metabolites (DMs), demonstrating unique metabolic profiles. Venn diagram showing the overlap between rheumatoid arthritis therapeutic targets and active component targets, revealing 119 common targets. (f) Kyoto encyclopedia of genes and genomes (KEGG) pathway and (g) gene ontology (GO) enrichment analysis of these common targets, highlighting the associated biological processes and pathways. RA, rheumatoid arthritis.

A total of 1289 metabolites from 11 categories were detected in the roots, stems, and leaves of the *I. seguinii* plant (Figure 3a). These include 344 lipids and lipid‐like molecules, 213 phenylpropanoids and polyketides, 121 benzene ring compounds, 110 oxygen‐containing organic compounds, 105 heterocyclic compounds, 104 organic acids and their derivatives, 29 alkaloids and their derivatives, 27 nitrogen‐containing organic compounds, 24 lignans and neolignans, and 20 nucleosides, nucleotides, and their analogs. The PCA results for both the experimental and quality control (QC) samples revealed that QC samples were tightly clustered at the center of the score plot under both positive (Figure 3b) and negative ion modes (Figure 3c), while the more dispersed distribution of root, stem, and leaf samples indicated significant differences in metabolite content within these tissues, a finding corroborated by the OPLS‐DA model (Figure S11a–c).

Ultimately, 137 differential metabolites (DM) were identified between leaves and stems (77 upregulated, 60 downregulated), 133 DMs between roots and leaves (54 upregulated, 79 downregulated), and 95 DMs between roots and stems (30 upregulated, 65 downregulated) (Figure S12a). There were 22 DMs common to all comparison groups, while 29 DMs were unique to the leaves and stems group, 16 to the roots and leaves group, and 26 to the roots and stems group (Figure S12b). A hierarchical clustering heatmap of these 207 differential metabolites showed that the samples within each group clustered together, indicating high intra‐group similarity. The clustering results of the rows indicated DMs with similar expression patterns (Figure 3d). These expression patterns could be divided into nine categories. Among them, 84 DMs had the highest relative content in leaves (expression modules 1, 2, 9), 50 DMs had the highest relative content in roots (expression modules 3, 4, 6), and 73 DMs had the highest relative content in stems (expression modules 5, 7, and 8) (Figure S12c).

### 3.9 Network pharmacology and molecular docking

**FIGURE 4:**
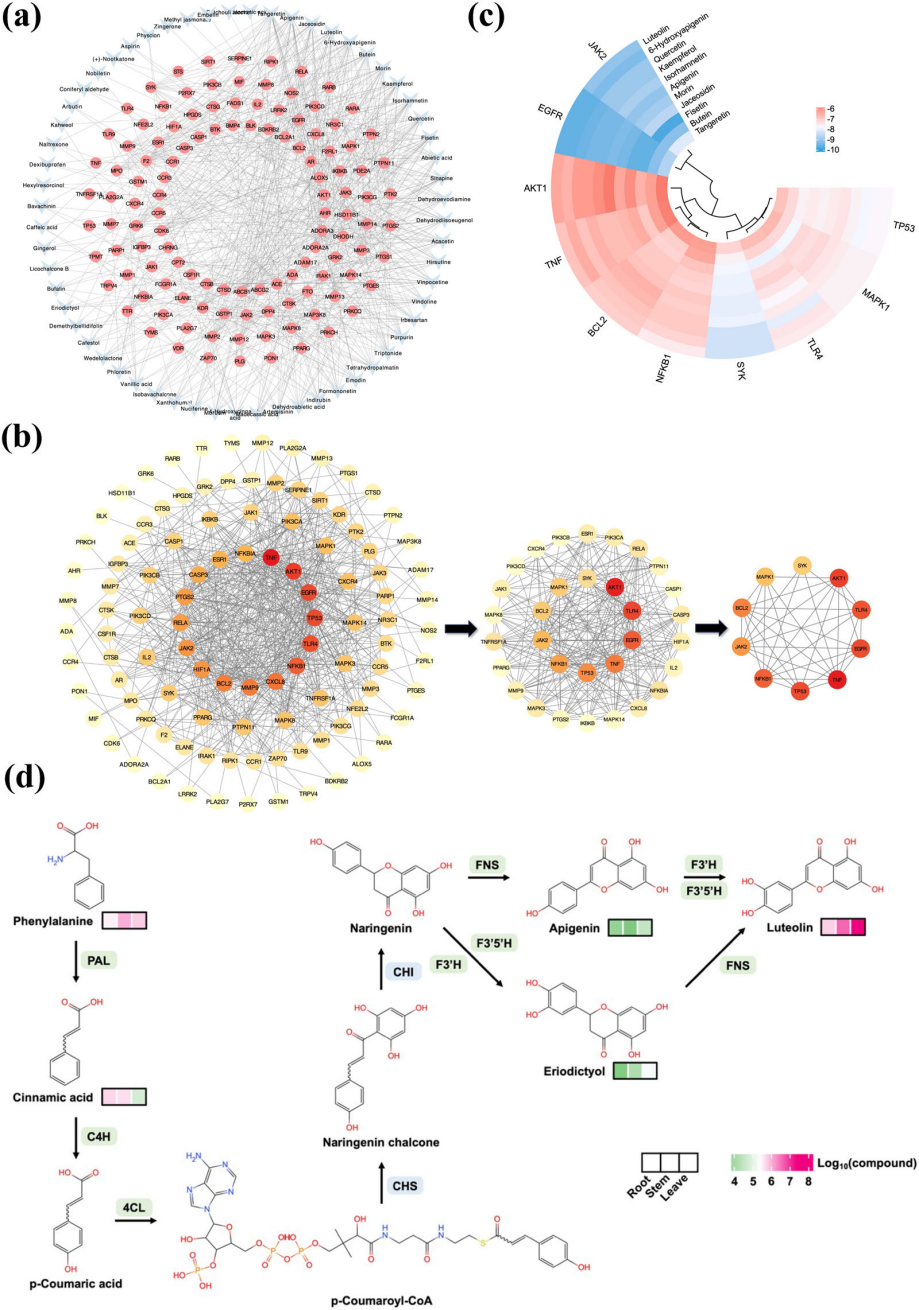
Detailed analysis of protein interactions and biosynthetic pathways of luteolin. (a) Component‐Target network illustrates the interactions between active ingredients in *I. seguinii* and therapeutic targets for rheumatoid arthritis. Blue nodes represent the active ingredients, while red nodes indicate intersection targets. Gray lines connect blue nodes to red nodes, showing interactions between the active ingredients and their targets. (b) Topological analysis of protein–protein interaction (PPI) network, highlighting key hub proteins and their interactions. (c) Heat map showing the binding energies of the main components with the hub targets, indicating potential therapeutic interactions. (d) Proposed biosynthetic pathway for luteolin, detailing the enzymatic steps and intermediate compounds involved. 4CL, 4‐coumarate: CoA ligase; C4H, cinnamic acid 4‐hydroxylase; CHI, chalcone isomerase; CHS, chalcone synthase; FNS, flavone synthase; PAL, phenylalanine ammonia‐lyase.

Based on the metabolite detection results from the roots, stems, and leaves of *I. seguinii*, 84 active compounds were identified with high bioavailability and possessing anti‐inflammatory, immunomodulatory, or immunosuppressive effects. These compounds adhere to at least four out of five drug‐likeness rules (Lipinski, Ghose, Veber, Egan, and Muegge). Using their SMILES codes as input data, target prediction on the SwissTargetPrediction website (http://www.swisstargetprediction.ch) identified 733 targets. Further searches using “rheumatoid arthritis” as a keyword in databases like DisGeNet, GeneCards, and OMIM yielded 829 potential therapeutic targets after filtering. A Venn diagram indicated an overlap of 119 RA therapeutic targets among the 733 interaction targets (Figure 3e). This relationship forms a “compound‐target” network with 666 edges. Hierarchical clustering and analysis identified 11 key bioactive compounds, predominantly flavonoids, except for Morin, a polyphenolic compound (Figure 4a).

The KEGG enrichment analysis indicated that the intersection targets were significantly enriched in the RA disease pathway (hsa05323), three immune response‐related pathways—Toll‐like receptor signaling pathway (hsa04620), T cell receptor signaling pathway (hsa04660), Th17 cell differentiation (hsa04659), three inflammation‐related immune response pathways—nuclear factor kappa‐light‐chain‐enhancer of activated B cells (NF‐κB) signaling pathway (hsa04064), chemokine signaling pathway (hsa04062), IL‐17 signaling pathway (hsa04657), and one immune regulation pathway—prolactin signaling pathway (hsa04917). This indicates that the therapeutic effects of the active ingredients on RA may be related to their regulation of the aforementioned pathways (Figure 3f).The results of the GO enrichment analysis showed that the intersection targets were significantly enriched in biological processes such as regulation of inflammatory response (GO:0050727), positive regulation of inflammatory response (GO:0050729), positive regulation of cytokine production (GO:0001819), leukocyte migration (GO:0050900), and cytokine‐mediated signaling pathway (GO:0019221). This suggests that the active ingredients may participate in regulating the human inflammatory response, playing roles in inhibiting cytokine secretion, cytokine‐mediated signaling pathways, and excessive migration of immune cells (Figure 3g).

**FIGURE 5:**
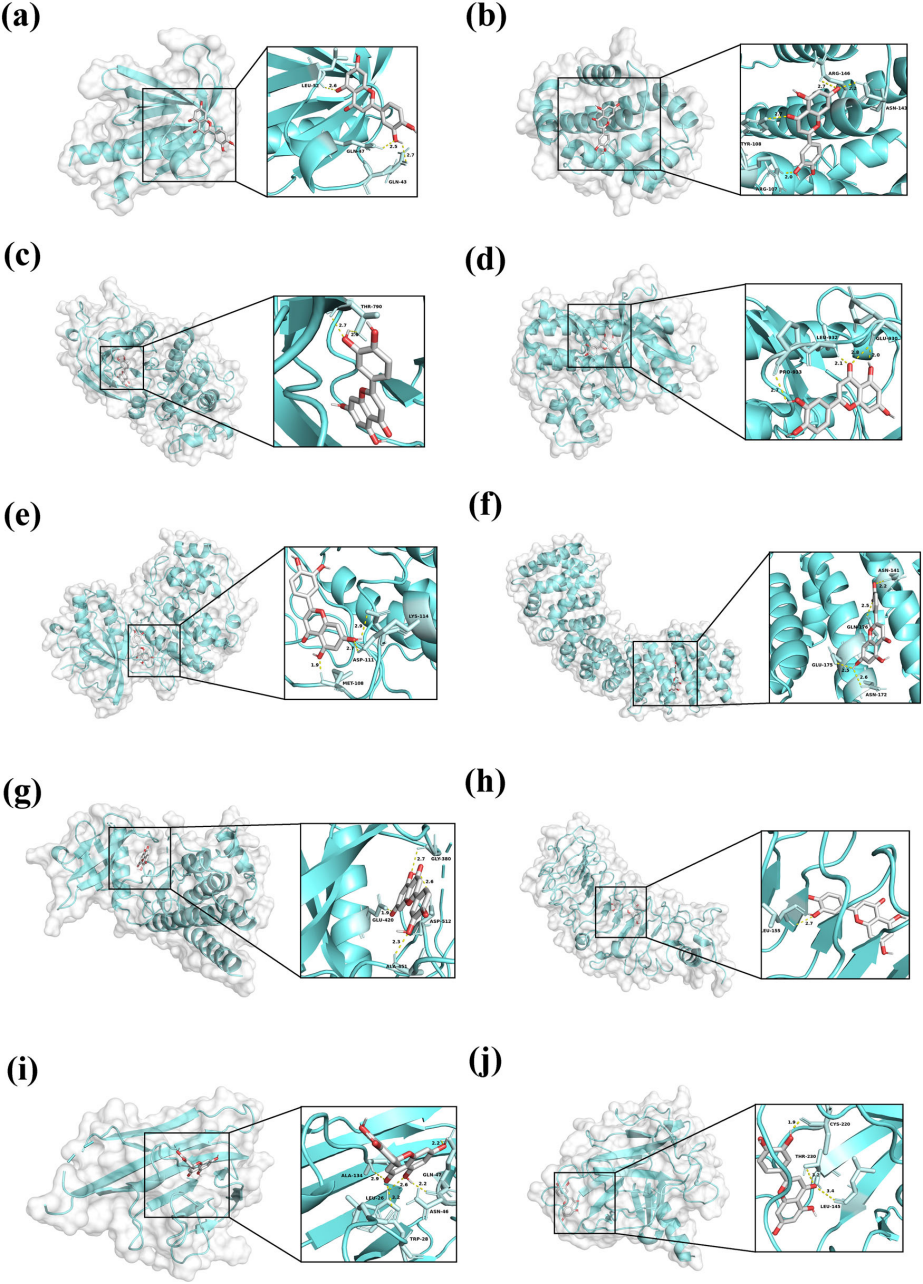
Three‐dimensional interactions of luteolin with key hub targets. This figure displays the docking results of luteolin with 10 critical targets: (a) *AKT1*, (b) *BCL2*, (c) *EGFR*, (d) *JAK2*, (e) *MAPK1*, (f) *NFKB1*, (g) *SYK*, (h) *TLR4*, (i) *TNF*, and (j) *TP53*. Each panel illustrates the interaction sites, with amino acid residues that bind to luteolin highlighted in light cyan. The names of the amino acid residues are labeled next to the binding sites, and the yellow dashed lines represent hydrogen bonds with distances indicated.

Further analyses in the STRING database constructed a PPI network of 106 proteins with 602 interactions, pinpointing highly connected core nodes crucial for network integrity and biological significance (Figure 4b). Based on degree centrality and intermediate centrality, the screened core targets included AKT serine/threonine kinase 1 (*AKT1*), toll‐like receptor 4 (*TLR4*), epidermal growth factor receptor (*EGFR*), tumor necrosis factor (*TNF*), tumor protein P53 (*TP53*), nuclear factor kappa B subunit 1 (*NFKB1*), janus kinase 2 (*JAK2*), BCL2 apoptosis regulator (*BCL2*), mitogen‐activated protein kinase 1 (*MAPK1*), and spleen‐associated tyrosine kinase (*SYK*). The affinity value of luteolin with two core therapeutic targets (*AKT1* and *TNF*) is greater than −7 kcal/mol, while its affinity value with the remaining eight core therapeutic targets (*TLR4*, *EGFR*, *TP53*, *NFKB1*, *JAK2*, *BCL2*, *MAPK1*, and *SYK*) is less than or equal to −7 kcal/mol (Figure 4c). Other key active ingredients (hesperetin, apigenin, isorhamnetin, wogonin, isorhamnetin, kaempferol, luteolin, myricetin, isorhamnetin, and quercetin) have an affinity value greater than −7 kcal/mol with at least three core therapeutic targets (Table S10). Furthermore, hydrogen bond visualization results indicate that luteolin is connected to each of the core therapeutic targets by three, four, two, four, three, four, three, one, four, and three hydrogen bonds, respectively. These results collectively suggest that luteolin can bind to the core therapeutic targets of RA and form structurally stable complexes (Figure 5).

### 3.10 Luteolin biosynthesis

This study detailed the biosynthetic pathway of flavonoids, focusing on luteolin, using data from the KEGG pathway database and existing literature. The biosynthesis begins with the conversion of phenylalanine to cinnamic acid by phenylalanine ammonia‐lyase (PAL) (Figure 4d). Cinnamic acid is then hydroxylated by cinnamic acid 4‐hydroxylase (C4H) to form p‐coumaric acid, which is linked with coenzyme A by 4‐coumarate: CoA ligase (4CL) to produce p‐coumaroyl‐CoA. CHS then catalyzes the formation of naringenin chalcone from p‐coumaroyl‐CoA and malonyl‐CoA. CHI converts naringenin chalcone to naringenin, a precursor for apigenin and eriodictyol, which are subsequently converted to luteolin by flavone synthase (FNS) and flavonol hydroxylases (flavanone 3′‐hydroxylase and flavanone 3′,5′‐hydroxylase).

Based on the qualitative and quantitative analysis of metabolites, this study determined the relative contents of five metabolites in the luteolin biosynthesis pathway: phenylalanine, cinnamic acid, apigenin, eriodictyol, and luteolin. As shown in Figure 4d, the relative content of apigenin, eriodictyol, and luteolin was highest in the leaves. In the stems, the relative content of eriodictyol and luteolin was higher than in the roots, while the relative content of apigenin was similar to that in the roots. Additionally, the relative content of phenylalanine was highest in the stems, and the relative content of cinnamic acid was highest in the roots.

**FIGURE 6:**
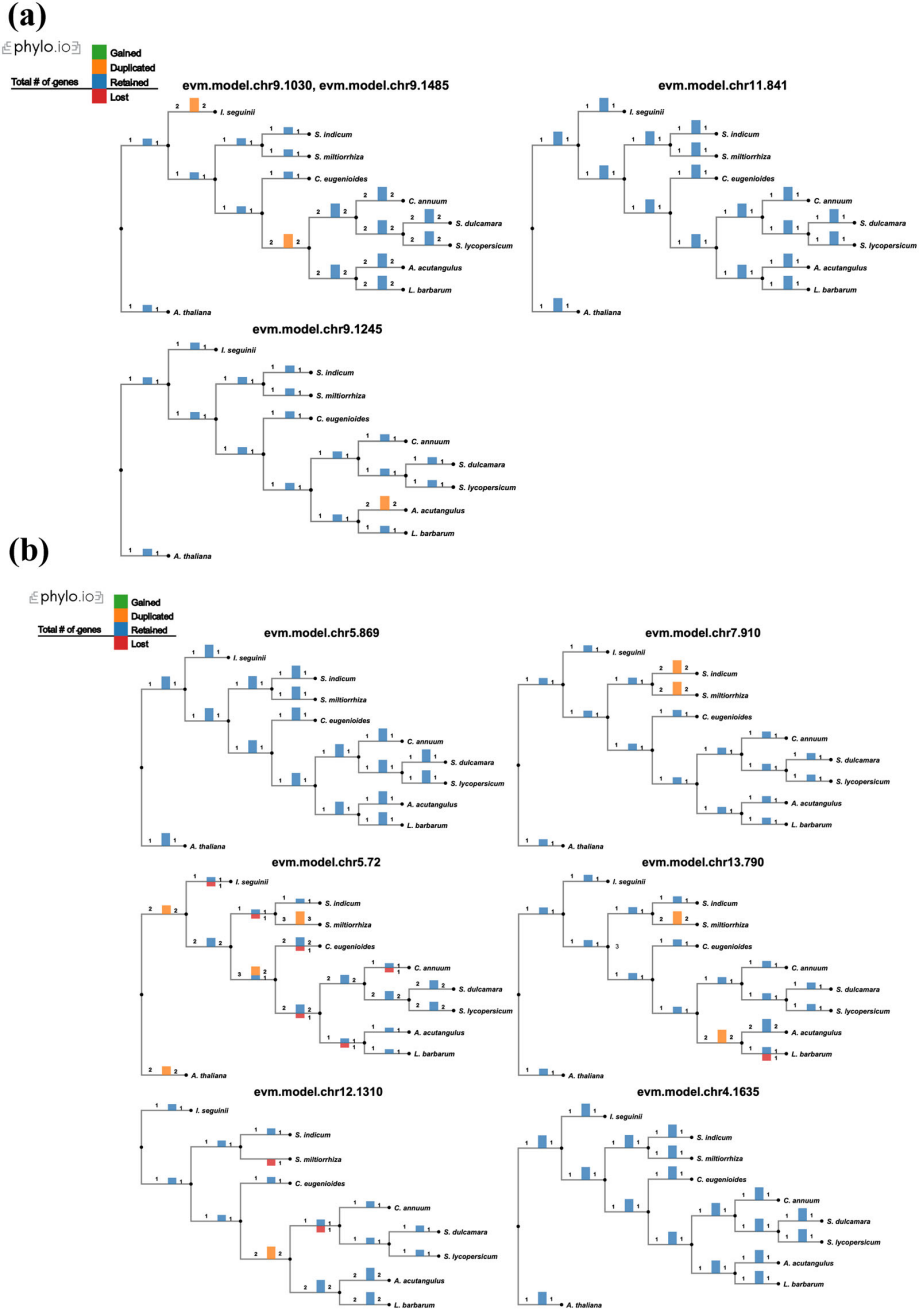
Chalcone synthase (CHS) and chalcone isomerase (CHI) hierarchical orthologous groups (HOGs) among 10 species. The yellow bars indicate gene duplication, the red bars indicate gene loss, and the blue bars represent genes that have neither gained nor lost.

From the above, it can be concluded that the rate‐limiting steps identified are the CHS‐catalyzed synthesis of naringenin chalcone and its isomerization by CHI. The results showed that three HOGs contained CHS genes, and six HOGs contained CHI genes. *I seguinii* acquired new CHS genes (evm.model.chr5.1279 and evm.model.chr5.1283) and also underwent CHS gene duplication (evm.model.chr9.1030 and evm.model.chr9.1485) without experiencing any loss (Table S11). The increase in CHS gene number may indicate an enhanced capacity for naringenin chalcone synthesis (Figure 6a). In contrast, the CHI gene in *I. seguinii* was lost, a phenomenon also observed in *C. arabica*, *C. annuum*, *L. barbarum*, and *S. miltiorrhiza* (Figure 6b).

## 4 DISCUSSION

To fill the genomic gap in the genus *Iodes* and even the order *Icacinales*, we utilized PacBio HiFi and Hi‐C sequencing data to achieve the first chromosome‐level assembly of the nuclear genome of *I*. *seguinii*. During the assembly process, the initial contigs sequences were assembled using PacBio HiFi sequencing data, resulting in an N50 length of 9.71 Mb. Subsequently, based on the alignment results of Hi‐C sequencing data, the contigs sequences were anchored to 13 chromosomes, with a total length of 273.58 Mb, which is very close to the estimated length (approximately 95% of the estimated length). The assembly quality of the chromosomes was evaluated from four aspects: BUSCO, LAI, transcriptome sequencing data mapping rate, and PacBio HiFi sequencing coverage. The results consistently indicated that the assembly quality, completeness, and continuity of the chromosomes are excellent. On this basis, various functional elements in the nuclear genome were annotated, resulting in the annotation of 115.28 Mb of repetitive sequences (covering 42.14% of the nuclear genome), 1062 RNA genes, and 25,270 protein‐coding genes (BUSCO evaluation result: 93.74%). The high‐quality assembly and annotation results lay a solid foundation for subsequent analysis of evolutionary processes, mining of functional genes, and exploration of biosynthetic pathways of active ingredients, providing valuable genomic resources for genetic improvement and synthetic biology research of active ingredients.

In addition to assembling and annotating the nuclear genome, we completed the de novo assembly and annotation of the chloroplast genome based on Illumina sequencing data. The chloroplast genome of *I*. *seguinii* is relatively large, at 150,599 bp, with the four components—LSC region, SSC region, IRA, and IRB—being 84,147, 18,932, 23,760, and 23,760 bp in length, respectively. The total length of this genome and the lengths of its components are very similar to those of the chloroplast genome of *I. cirrhosa*. Using the annotated information of the *I. cirrhosa* chloroplast genome as a reference, we annotated the chloroplast genome of *I*. *seguinii*, resulting in the annotation of 121 genes, including some commonly used DNA barcodes such as *rbcL*, *matK*, *psbA*, *trnH*, *accD*, and *ycf1*. We performed PCR amplification for *rbcL*, *matK*, and the noncoding region between *psbA* and *trnH* and used their sequencing results for species identification. The results showed that *matK* can be used as a basis for the identification of *I*. *seguinii* with better species identification efficacy than *rbcL* and *psbA‐trnH*.

To determine the phylogenetic relationship between *I*. *seguinii* and other *Lamiales* plants and to analyze the evolutionary phenomena in its adaptive evolution, we conducted an evolutionary analysis based on the nuclear genomes of *I*. *seguinii*, two *Lamiales* species, one *Gentianales* species, and five *Solanales* species. The results showed that *I*. *seguinii* is positioned at the base of the *Lamiales* clade, indicating it is a basal *Lamiales* species. The divergence between *I*. *seguinii* and the core *Lamiales* (*Gentianales*, *Lamiales*, *Solanales*) occurred approximately 117.8 million years ago, consistent with previous findings based on chloroplast genomes or pollen traits (Alawfi & Alzahrani, 2023; Stull et al., 2015). Additionally, 1566 gene families in *I*. *seguinii* have expanded, 1663 gene families have contracted, and 156 genes are under positive selection. Genes within significantly expanded or contracted gene families are related to the synthesis of secondary metabolites, while genes under positive selection are associated with DNA damage repair. The expansion of gene families in *I*. *seguinii* may be attributed to polyploidization events. According to the Ks distribution, *I. seguinii*, along with *S. dulcamara* and *S. indicum*, experienced a whole‐genome duplication event between 154.4 and 162.7 million years ago. After divergence, *I. seguinii* underwent another independent whole‐genome duplication event between 88.2 and 92.9 million years ago, whereas *S. dulcamara* and *S. indicum* experienced a whole‐genome duplication event together with *L. barbarum*. Consequently, there is no one‐to‐one syntenic relationship between the nuclear genomes of *I. seguinii* and either *S. dulcamara* or *S. indicum*.

Based on the metabolite detection results from the roots, stems, and leaves of *I. seguinii*, this study identified a total of 84 active components. Using disease target information from public databases and target prediction results, 119 targets were identified for RA treatment. Among these, the core therapeutic targets closely related to the development and progression of RA are *AKT1*, *TLR4*, *EGFR*, *TNF*, *TP53*, *NFKB1*, *JAK2*, *BCL2*, *MAPK1*, and *SYK*. Research indicates that the expression levels of *BCL2*, *TP53*, and *EGFR* are significantly elevated in patients with RA (Alawfi & Alzahrani, 2023; Taghadosi et al., 2021; Yuan et al., 2013). BCL2, an apoptosis inhibitor, helps maintain the integrity of mitochondrial structures in affected joints. TP53, a transcription factor, alleviates inflammation by interfering with the NF‐κB and MAPK signaling pathways and reducing the expression of inflammatory factors. EGFR, a transmembrane glycoprotein, promotes the proliferation of synovial fibroblasts and the secretion of cytokines while also inhibiting the formation and differentiation of osteoclasts. Targeting EGFR can alleviate RA symptoms. Among the other core therapeutic targets, both SYK and JAK2 are non‐receptor tyrosine kinases; SYK regulates the formation and secretion of cytokines, osteoclast maturation, and platelet aggregation, while JAK2 is crucial for hematopoiesis. Reducing the activity of these enzymes can help improve the condition (Cooper et al., 2023; Ma et al., 2016; Roskoski, 2022). AKT1 and MAPK1 are serine/threonine kinases; AKT1 is a central node in the phosphoinositide 3‐kinase signaling pathway, which is associated with abnormal proliferation of fibroblast‐like synoviocytes and synovitis, as well as osteoclast formation and differentiation. MAPK1 plays a key role in regulating the secretion of pro‐inflammatory cytokines and is related to joint inflammation and damage. TLR4, a pattern recognition receptor, is activated by specific exogenous substances or endogenous molecules, triggering innate immune and inflammatory responses. However, excessive activation leads to the production of large amounts of inflammatory factors (Zhang et al., 2022). TNF is a pro‐inflammatory cytokine, and antagonists developed against it are powerful tools for treating RA (Balkwill, 2009).

The subsequent topological analysis of the “compound‐target” network revealed that the key active components in *I. seguinii* are 10 flavonoids (hesperetin, apigenin, luteolin, chrysin, baicalein, morin, kaempferol, isorhamnetin, fisetin, quercetin) and one polyphenol (scutellarin). These compounds all possess anti‐inflammatory activity and can stably bind to core therapeutic targets for RA to exert their therapeutic effects (Conti et al., 2021; Kim, 2023; X. R. Liu et al., 2024; Nam et al., 2013; Periferakis et al., 2022; Shina et al., 2009; Singaravelu et al., 2021; M. Tang et al., 2022; Y. Wang et al., 2022; G. Yang, Xia, et al., 2021). Additionally, studies have shown that apigenin, fisetin, and quercetin can inhibit the proliferation of fibroblast‐like synoviocytes, while kaempferol, isorhamnetin, and scutellarin can inhibit their migration and invasion. Hesperetin and luteolin can downregulate the levels of pro‐inflammatory cytokines (Lee et al., 2009; Pan et al., 2018; L. Yang, Cao, et al., 2021). Since the abnormal proliferation of fibroblast‐like synoviocytes and the excessive secretion of pro‐inflammatory cytokines are the main pathological changes in the affected areas of RA, these active components may help alleviate the symptoms of RA and hold promise as the basis for developing new therapeutic drugs for the disease.

Before luteolin was detected in *I. seguinii* in this study, this flavonoid had already been found in various Chinese medicinal herbs (such as *Cleome rutidosperma*, *Crocus sativus*, *Cyperus rotundus*, *Matricaria chamomilla*, *Ocimum basilicum*, *Perilla frutescens*, *Portulaca oleracea*, *Punica granatum*, and *Zingiber officinale*) as well as in fruits and vegetables (such as celery, parsley, broccoli, onion, cabbage, and apple) (Taban et al. 2023). There are also two key rate‐limiting enzymes in its biosynthetic pathway: CHS and CHI. CHS catalyzes the condensation reaction between one molecule of coumaroyl‐CoA and three molecules of malonyl‐CoA, producing naringenin chalcone; CHI catalyzes the rapid isomerization of naringenin chalcone to naringenin (Yin et al., 2019). The CHS gene sequence is highly conserved across different plants, but the number of copies and the chromosomes containing this gene vary significantly. For example, the nuclear genome of parsley contains only one CHS gene, while that of snapdragon contains 14, and rice has 27. The CHS gene is present on only one chromosome in *A. thaliana*, but it is found on seven chromosomes in soybean. Using sequence similarity comparison, this study identified six CHS genes in *I. seguinii*, distributed across three chromosomes. Subsequently, by analyzing HOGs, we found that the CHS gene family in *I. seguinii* expanded through gene duplication and the formation of new genes. This expansion, likely due to polyploidization events, may contribute to the synthesis and accumulation of luteolin in *I. seguinii*. Such expansion of key gene families is also common in other Chinese medicinal herbs (Kang et al., 2020; J. Wang et al., 2021; Xu et al., 2020). In contrast, the CHI gene family in *I. seguinii* contracted due to gene loss, a phenomenon also observed in *C. arabica*, *C. annuum*, *L. barbarum*, and *S. miltiorrhiza*.

In summary, we assessed the effectiveness of using DNA barcoding technology to identify *I. seguinii* and achieved high‐precision assembly and annotation of its nuclear and chloroplast genomes by integrating advanced sequencing and genome assembly technologies such as Illumina, PacBio HiFi, and Hi‐C data. Through genomic evolutionary analysis, this study successfully constructed the phylogenetic tree of *I. seguinii*, elucidating its evolutionary relationships with other Lamiaceae species. Furthermore, by combining untargeted analysis and network pharmacology, this study identified components in *I. seguinii* with potential therapeutic effects on RA, focusing specifically on the biosynthesis pathway of the key compound luteolin and the changes in its key gene families. These findings provide a scientific basis for the development and application of *I. seguinii*, but there are still gaps in the breadth of research. Future studies need to delve deeper into areas such as genomics, transcriptomics, and metabolomics.

## AUTHOR CONTRIBUTIONS

**Xun Gong**: Conceptualization; funding acquisition; resources; writing—original draft. **Hantao Zhang**: Data curation; software; writing—review and editing. **Yinluo Guo**: Visualization; writing—review and editing. **Shaoshuai Yu**: Methodology; resources; writing—review and editing. **Min Tang**: Project administration; writing—review and editing.

## CONFLICT OF INTEREST STATEMENT

The authors declare no conflicts of interest.

## Supporting information



Figure S1 Comprehensive species identification through *rbcL, psbA‐trnH* and *matK* gene amplification.

Figure S2 Bioinformatics workflow for genomic assembly and annotation of *I. Seguinii*.

Figure S3 Flowchart for assembling and annotating the chloroplast genome and surveying the nuclear genome

Figure S4 Flowchart for integrated network pharmacology analysis of metabolite assay data.

Figure S5 Overview of sequencing data and genomic Analysis.

Figure S6 Distribution of repeats in the nuclear genome of *I*. *seguinii*.

Figure S7 GO and KEGG enrichment analysis of genes in the gene families of *I. seguinii* undergoing significant (a, b) expansion, (c, d) contraction and (e, f) positive selection.

Figure S8 Chromosome‐wise self‐synteny of *I. seguinii*, illustrating the syntenic relationships within its chromosomes

Figure S9 Collinear relationships among *I. seguinii*, *S. dulcamara*, and *S. indicum*.

Figure S10 Synteny map of *I. seguinii* pseudochromosomes compared to contigs of other species reveals a close relationship, with significant portions of the chromosomes conserved between the species.

Figure S11 Multivariate analysis and clustering of DMs.

Figure S12 Comprehensive Analysis of DMs and FCM Clustering.

Table S1 Identification of species using *rbcL*, *psbA‐trnH* and *matK* gene amplification with corresponding forward (F) and reverse (R) Primers.

Table S2 Detailed results from the BOLD for accurate species identification using DNA barcoding.

Table S3 Results of sequence alignment for *psbA‐trnH* against NT database.

Table S4 Chromosome assembly and global statistics for *I. seguinii*.

Table S5 Length statistics of different types of repeats in the nuclear genome of *I. seguinii*

Table S6 Number and length statistics of RNA genes in nuclear genome of *I. seguinii*

Table S7 Genome annotation evaluation using BUSCO analysis

Table S8 Functional annotation of *I. seguinii* protein‐coding genes

Table S9 Classification of genes encoded by the chloroplast genome of *I*. *seguinii*

Table S10 Molecular docking results of the main components in *I*. *seguinii* with the hub targets

Table S11 CHS and CHI genes in the nuclear genome of *I*. *seguinii*

## Data Availability

The whole genome sequence data reported in this paper have been deposited in the Genome Warehouse in the National Genomics Data Center, Beijing Institute of Genomics, Chinese Academy of Sciences/China National Center for Bioinformation, under accession number GWHERDR00000000 that is publicly accessible at https://ngdc.cncb.ac.cn/gwh.
