# Supplementary material for: Chromosome‐level genome assembly of Iodes seguinii and its metabonomic implications for rheumatoid arthritis treatment
Source: Plant Genome. 2024 Nov 27;18(1):e20534. doi: 10.1002/tpg2.20534 (PMC11729983; doi:10.1002/tpg2.20534)
Supplement: Supplementary file 4 [file TPG2-18-e20534-s004.docx]

**Table S11 CHS and CHI genes in the nuclear genome of *I*. *seguinii***

| **Gene ID** | **Description** | **Function** |
| --- | --- | --- |
| evm.model.chr5.1279 | CHS | Chalcone synthase |
| evm.model.chr5.1283 | CHS | Chalcone synthase |
| evm.model.chr9.1485 | CHS | Chalcone synthase |
| evm.model.chr11.841 | CHS | Chalcone synthase |
| evm.model.chr9.1245 | CHS | Chalcone synthase |
| evm.model.chr9.1030 | CHS | Chalcone synthase |
| evm.model.chr5.869 | CHI | Chalcone isomerase |
| evm.model.chr7.910 | CHI | Chalcone isomerase |
| evm.model.chr5.72 | CHI | Chalcone isomerase |
| evm.model.chr13.790 | CHI | Chalcone isomerase |
| evm.model.chr12.1310 | CHI | Chalcone isomerase |
| evm.model.chr4.1635 | CHI | Chalcone isomerase |
