# Supplementary material for: Chromosome‐level genome assembly of Iodes seguinii and its metabonomic implications for rheumatoid arthritis treatment
Source: Plant Genome. 2024 Nov 27;18(1):e20534. doi: 10.1002/tpg2.20534 (PMC11729983; doi:10.1002/tpg2.20534)
Supplement: Supplementary file 7 [file TPG2-18-e20534-s007.docx]

## Table S4 Chromosome assembly and global statistics for *I. seguinii*.

| **Chr** | **Chr size (bp)** | **No. of Contigs** | **GC content (%)** | **Repeats (%)** | **CDS length (bp)** | **Coding  sequences (%)** | **CDS count** |  |
| --- | --- | --- | --- | --- | --- | --- | --- | --- |
|  |  |  |  |  |  |  |  |  |
| Chr01 | 36,592,283 | 3 | 32.98 | 27.22 | 5,443,812 | 14.88 | 23,276 |  |
| Chr02 | 23,934,564 | 5 | 32.69 | 50.81 | 2,248,578 | 9.39 | 8,410 |  |
| Chr03 | 22,182,189 | 12 | 32.84 | 45.97 | 2,283,956 | 10.30 | 9,611 |  |
| Chr04 | 22,125,846 | 5 | 33.00 | 41.64 | 2,449,200 | 11.07 | 10,354 |  |
| Chr05 | 21,383,588 | 9 | 32.08 | 56.23 | 1,866,917 | 8.73 | 7,203 |  |
| Chr06 | 20,897,580 | 3 | 32.63 | 52.66 | 1,841,471 | 8.81 | 7,380 |  |
| Chr07 | 20,630,438 | 5 | 32.05 | 45.89 | 2,173,852 | 10.54 | 8,363 |  |
| Chr08 | 19,938,035 | 4 | 32.58 | 38.44 | 2,366,609 | 11.87 | 9,956 |  |
| Chr09 | 18,721,715 | 5 | 33.34 | 38.91 | 2,281,819 | 12.19 | 9,619 |  |
| Chr10 | 18,272,200 | 2 | 32.90 | 28.29 | 2,512,644 | 13.75 | 10,599 |  |
| Chr11 | 17,511,481 | 9 | 32.96 | 47.65 | 1,905,425 | 10.88 | 7,783 |  |
| Chr12 | 16,465,758 | 4 | 32.69 | 37.23 | 2,004,404 | 12.17 | 8,093 |  |
| Chr13 | 14,922,929 | 7 | 32.57 | 44.00 | 1,608,931 | 10.78 | 6,136 |  |
| Total 13 Chr | 273,578,606 | 73 | 32.73 | 42.10 | 30,987,618 | 11.33 | 126,783 |  |
