# Supplementary material for: Chromosome‐level genome assembly of Iodes seguinii and its metabonomic implications for rheumatoid arthritis treatment
Source: Plant Genome. 2024 Nov 27;18(1):e20534. doi: 10.1002/tpg2.20534 (PMC11729983; doi:10.1002/tpg2.20534)
Supplement: Supplementary file 10 [file TPG2-18-e20534-s010.docx]

**Figure S2 Bioinformatics workflow for genomic assembly and annotation of *I. Seguinii***. The process begins with PacBio HiFi reads, followed by various stages including assembly using Hifiasm and gfatools, quality evaluation with BUSCO, scaffolding, and repeat analysis. Gene prediction and functional annotation are conducted using multiple tools like Augustus and InterProScan. The workflow also includes phylogenetic analysis with IQ-tree and investigation of gene family dynamics and whole genome duplication events using tools like CAFE5 and WGDI.
