# Supplementary material for: Chromosome‐level genome assembly of Iodes seguinii and its metabonomic implications for rheumatoid arthritis treatment
Source: Plant Genome. 2024 Nov 27;18(1):e20534. doi: 10.1002/tpg2.20534 (PMC11729983; doi:10.1002/tpg2.20534)
Supplement: Supplementary file 13 [file TPG2-18-e20534-s013.docx]

**Table S9 Classification of genes encoded by the chloroplast genome of *I*. *seguinii***

| **Category** | **Gene groups** | **Gene names** |
| --- | --- | --- |
| Self-replication | Transfer RNAs | *trnH-GTG, trnQ-TTG, trnS-GCT, trnR-TCT, trnC-GCA, trnD-GTC, trnY-GTA, trnE-TTC, trnT-GGT, trnS-TGA, trnG-GCC, trnM-CAT (×2), trnS-GGA, trnT-TGT, trnF-GAA, trnW-CCA, trnP-TGG, trnI-CAT (×2), trnL-CAA, trnV-GAC* (×2)*, trnR-ACG* (×2)*, trnN-GTT* (×2)*, trnL-TAG* |
|  | Ribosomal RNAs | *rrn16S* (×2)*, rrn23S* (×2)*, rrn4.5S* (×2)*, rrn5S* (×2) |
|  | Large subunit of ribosome | *rpl14, rpl2* (×2)^a^*, rpl20, rpl22, rpl23* (×2)*, rpl32, rpl33, rpl36* |
|  | Small subunit of ribosome | *rps11, rps12 (×2)^a^, rps14, rps15, rps16^a^, rps18, rps19, rps2, rps3, rps4, rps7 (×2), rps8* |
|  | DNA dependent RNA polymerase | *rpoA, rpoB, rpoC1*^a^*, rpoC2* |
| Photosynthesis | Subunits of photosystem I | *psaA, psaB, psaC, psaI, psaJ* |
|  | Subunits of photosystem II | *psbA, psbB, psbC, psbD, psbE, psbF, psbH, psbI, psbJ, psbK, psbM, psbN, psbT, psbZ, ycf3*^b^ |
| NADH dehydrogenase | Subunits of NADH-dehydrogenase | *ndhA^a^, ndhB* (×2)^a^*, ndhC, ndhD, ndhE, ndhF, ndhG, ndhH, ndhI, ndhJ, ndhK* |
|  | Subunits of cytochrome b/f complex | *petA, petB*^a^*, petD*^a^*, petG, petL, petN* |
|  | Subunits of ATP synthase | *atpA, atpB, atpE, atpF*^a^, *atpH, atpI* |
|  | Subunit of rubisco | *rbcL* |
| Other genes | Maturase | *matK* |
|  | Envelop membrane protein | *cemA* |
|  | Subunit of Acetyl-CoA-carboxylase | *accD* |
|  | c-type cytochrom synthesis gene | *ccsA* |
|  | Protease | *clpP*^b^ |
|  | Translational initiation factor | *infA* |
|  | Conserved open reading frames | *ycf1, ycf15 (×4), ycf2 (×2), ycf4* |

* (×2) indicates that the gene has 2 copies, (×4) indicates that the gene has 4 copies, "a" indicates that the gene contains 1 intron, and "b" indicates that the gene contains 2 introns.
