# Supplementary material for: Chromosome‐level genome assembly of Iodes seguinii and its metabonomic implications for rheumatoid arthritis treatment
Source: Plant Genome. 2024 Nov 27;18(1):e20534. doi: 10.1002/tpg2.20534 (PMC11729983; doi:10.1002/tpg2.20534)
Supplement: Supplementary file 16 [file TPG2-18-e20534-s016.docx]

**Table S1 Identification of species using *rbcL*, *psbA-trnH* and *matK* gene amplification with corresponding forward (F) and reverse (R) Primers.**

| **Locus** | **Primer sets** | **Reaction condition** |  |
| --- | --- | --- | --- |
| *rbc*L | F:ATGTCACCACAAACAGAGACTAAAGC R:GTAAAATCAAGTCCACCGCG | 95℃ 3 min 95℃ 15 s, 55℃ 30s, 72℃ 1 min, 35 cycles 72℃ 5 min |  |
|  |  |  |  |
| *psbA-trnH* | F:GTTATGCATGAACGTAATGCTC R:CGCGCATGGTGAATTCACAATCC | 95℃ 3 min 95℃ 15 s, 52℃ 30s, 72℃ 1 min (35 cycles) 72℃ 5 min |  |
| *mat*K | F1:CACAGATTCTTCTTC R1:TTCTGCATATACGCC | 95℃ 3 min 95℃ 15 s, 52℃ 30s, 72℃ 1 min, 35 cycles 72℃ 5 min |  |
|  | F2:TTGCATTTATTACGATTCTTTCTCC R2:CTATAATAATGAAAAAGATTTCTGC |  |  |
|  | F3:GTTCAAACTATTCGCTACTGG R3:TTCTGGCACACGAAAGTCG |  |  |
|  | F4:CTCCTCCTTGCATTTATTACG R4:GAGGATCCACTATAATAATG |  |  |
