# Supplementary material for: Chromosome‐level genome assembly of Iodes seguinii and its metabonomic implications for rheumatoid arthritis treatment
Source: Plant Genome. 2024 Nov 27;18(1):e20534. doi: 10.1002/tpg2.20534 (PMC11729983; doi:10.1002/tpg2.20534)
Supplement: Supplementary file 17 [file TPG2-18-e20534-s017.docx]

**Figure S9 Collinear relationships among *I. seguinii*, *S. dulcamara*, and *S. indicum***. This figure illustrates the syntenic blocks shared between the genomes of *I. seguinii*, *S. dulcamara*, and *S. indicum*, highlighting the evolutionary genomic structures and the extent of conserved gene order among these species.
