# Supplementary material for: Chromosome‐level genome assembly of Iodes seguinii and its metabonomic implications for rheumatoid arthritis treatment
Source: Plant Genome. 2024 Nov 27;18(1):e20534. doi: 10.1002/tpg2.20534 (PMC11729983; doi:10.1002/tpg2.20534)
Supplement: Supplementary file 18 [file TPG2-18-e20534-s018.docx]

**Figure S3 Flowchart for assembling and annotating the chloroplast genome and surveying the nuclear genome**
