# Supplementary material for: Chromosome‐level genome assembly of Iodes seguinii and its metabonomic implications for rheumatoid arthritis treatment
Source: Plant Genome. 2024 Nov 27;18(1):e20534. doi: 10.1002/tpg2.20534 (PMC11729983; doi:10.1002/tpg2.20534)
Supplement: Supplementary file 19 [file TPG2-18-e20534-s019.docx]

**Table S6 Number and length statistics of RNA genes in nuclear genome of *I. seguinii***

| **Type** |  | **Number** | **Average length (bp)** | **Total length (bp)** | **% of genome** |
| --- | --- | --- | --- | --- | --- |
| miRNA |  | 126 | 7.7 | 966 | 0.0004 |
| tRNA |  | 390 | 75.1 | 29,302 | 0.0107 |
| rRNA | 8S | 121 | 115 | 13,916 | 0.0051 |
|  | 18S | 2 | 1728 | 3,456 | 0.0013 |
|  | 28S | 2 | 4969 | 9,938 | 0.0036 |
|  | total | 125 | 218.5 | 27,310 | 0.01 |
| snRNA | CD-box | 312 | 106.9 | 33,359 | 0.0122 |
|  | HACA-box | 35 | 126 | 4,409 | 0.0016 |
|  | splicing | 74 | 138.6 | 10,258 | 0.0037 |
|  | total | 421 | 114.1 | 48,026 | 0.0176 |
