# Supplementary material for: Chromosome‐level genome assembly of Iodes seguinii and its metabonomic implications for rheumatoid arthritis treatment
Source: Plant Genome. 2024 Nov 27;18(1):e20534. doi: 10.1002/tpg2.20534 (PMC11729983; doi:10.1002/tpg2.20534)
Supplement: Supplementary file 21 [file TPG2-18-e20534-s021.docx]

**Table S8 Functional annotation of *I. seguinii* protein-coding genes**

| **Database** | **Annotated number** | **Annotated Percent (%)** |
| --- | --- | --- |
| NR | 23,905 | 94.60 |
| Swiss-Prot | 5,176 | 20.48 |
| eggNOG | 23,299 | 92.20 |
| KEGG | 9,777 | 38.69 |
| InterPro | 19,099 | 75.58 |
| Pfam | 19,800 | 78.35 |
| GO | 16,992 | 67.24 |
| Annotated | 23,998 | 94.97 |
| Total | 25,270 | - |
