# Supplementary material for: Chromosome‐level genome assembly of Iodes seguinii and its metabonomic implications for rheumatoid arthritis treatment
Source: Plant Genome. 2024 Nov 27;18(1):e20534. doi: 10.1002/tpg2.20534 (PMC11729983; doi:10.1002/tpg2.20534)
Supplement: Supplementary file 23 [file TPG2-18-e20534-s023.docx]

**Figure S11 Multivariate analysis and clustering of DMs.** The score plots of OPLS-DA pairwise comparisons of DMs. (a) root vs. stem. (b) root vs. leaf. (c) stem vs. leaf.
