# Supplementary material for: Chromosome‐level genome assembly of Iodes seguinii and its metabonomic implications for rheumatoid arthritis treatment
Source: Plant Genome. 2024 Nov 27;18(1):e20534. doi: 10.1002/tpg2.20534 (PMC11729983; doi:10.1002/tpg2.20534)
Supplement: Supplementary file 1 — Figure S1 Comprehensive species identification through rbcL, psbA‐trnH and matK gene amplification. [file TPG2-18-e20534-s020.docx]

**Figure S1 Comprehensive species identification through *rbcL, psbA-trnH* and *matK* gene amplification.** (a) *rbcL* and (b) *psbA-trnH* gene amplification analysis in three samples using a single primer set. (c) *matK* gene amplification analysis in three samples across four distinct primer sets. IS, Iodes seguinii.
