# Supplementary material for: Chromosome‐level genome assembly of Iodes seguinii and its metabonomic implications for rheumatoid arthritis treatment
Source: Plant Genome. 2024 Nov 27;18(1):e20534. doi: 10.1002/tpg2.20534 (PMC11729983; doi:10.1002/tpg2.20534)
Supplement: Supplementary file 4 — Figure S4 Flowchart for integrated network pharmacology analysis of metabolite assay data. [file TPG2-18-e20534-s011.docx]

**Figure S4 Flowchart for integrated network pharmacology analysis of metabolite assay data.** The diagram outlines the comprehensive workflow used for analyzing metabolite assay data through an integrated network pharmacology approach. It begins with mass spectral data processing, which is analyzed using various tools and databases. The analysis includes determining DMs, active compounds, putative targets, and therapeutic targets. These results are then cross-referenced to identify common targets, which are further analyzed to construct a compound-target-disease network and a PPI network. Subsequent steps involve identifying main active compounds and hub targets, followed by molecular docking studies and biosynthetic pathway construction to elucidate the pharmacological mechanisms.
