# Supplementary figures and images for: Chromosome‐level genome assembly of Iodes seguinii and its metabonomic implications for rheumatoid arthritis treatment
Source: Plant Genome. 2024 Nov 27;18(1):e20534. doi: 10.1002/tpg2.20534 (PMC11729983; doi:10.1002/tpg2.20534)

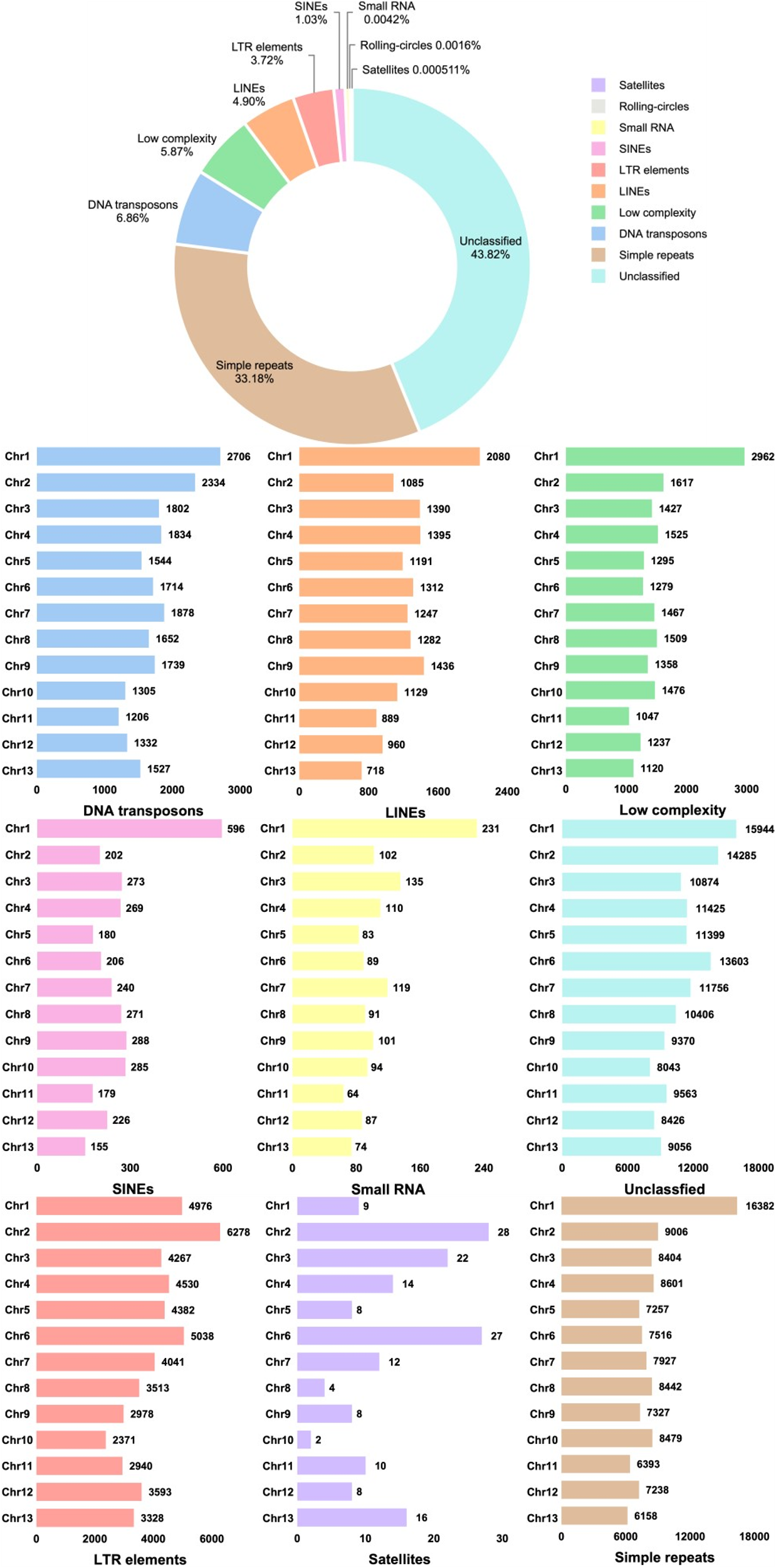
**Figure S6 Distribution of repeats in the nuclear genome of *I*. *seguinii.***

Supplement: Supplementary file 6 — Figure S6 Distribution of repeats in the nuclear genome of I. seguinii. [file TPG2-18-e20534-s008.docx]
