# Supplementary material for: Chromosome‐level genome assembly of Iodes seguinii and its metabonomic implications for rheumatoid arthritis treatment
Source: Plant Genome. 2024 Nov 27;18(1):e20534. doi: 10.1002/tpg2.20534 (PMC11729983; doi:10.1002/tpg2.20534)
Supplement: Supplementary file 7 — Figure S7 GO and KEGG enrichment analysis of genes in the gene families of I. seguinii undergoing significant (a, b) expansion, (c, d) contraction and (e, f) positive selection. [file TPG2-18-e20534-s002.docx]

**Figure S7 GO and KEGG enrichment analysis of genes in the gene families of *I. seguinii* undergoing significant (a, b) expansion, (c, d) contraction and (e, f) positive selection.**
