# Supplementary material for: Chromosome‐level genome assembly of Iodes seguinii and its metabonomic implications for rheumatoid arthritis treatment
Source: Plant Genome. 2024 Nov 27;18(1):e20534. doi: 10.1002/tpg2.20534 (PMC11729983; doi:10.1002/tpg2.20534)

**Figure S8 Chromosome-wise self-synteny of *I. seguinii*, illustrating the syntenic relationships within its chromosomes**


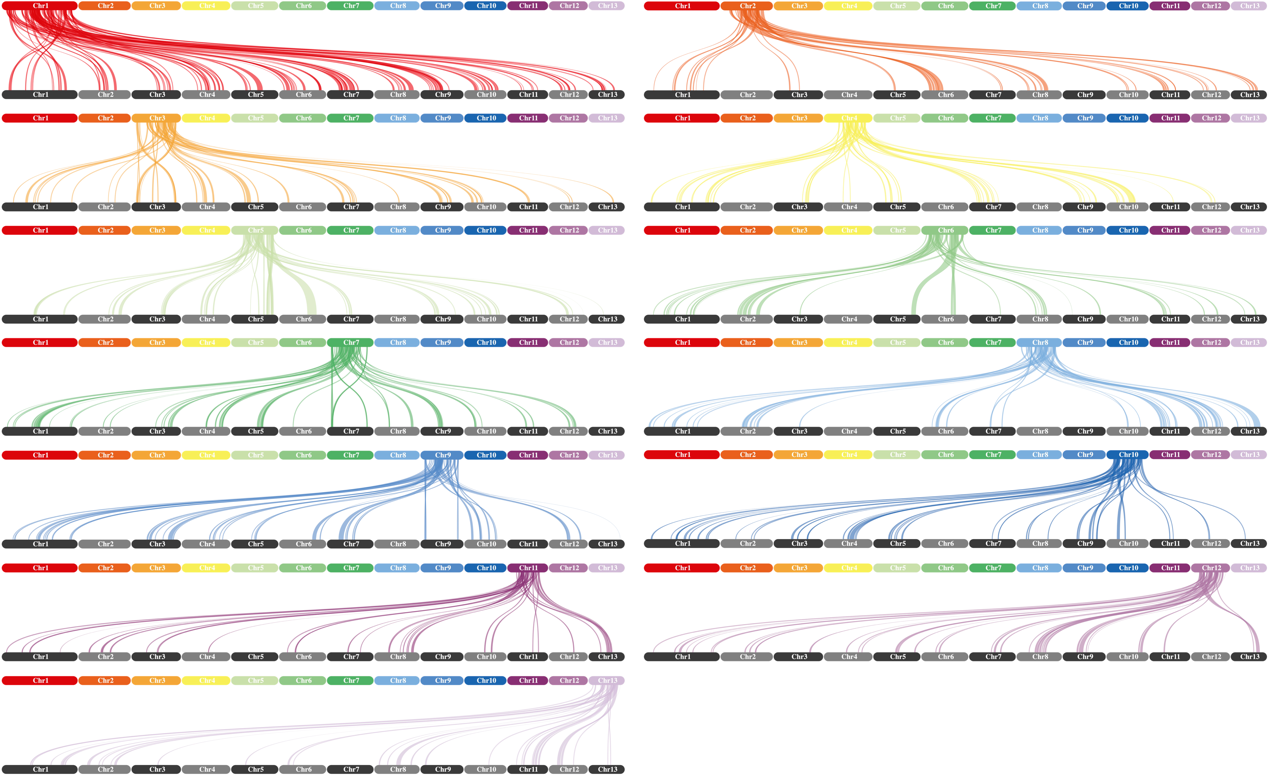

Supplement: Supplementary file 8 — Figure S8 Chromosome‐wise self‐synteny of I. seguinii, illustrating the syntenic relationships within its chromosomes [file TPG2-18-e20534-s014.docx]
