# Supplementary material for: Chromosome‐level genome assembly of Iodes seguinii and its metabonomic implications for rheumatoid arthritis treatment
Source: Plant Genome. 2024 Nov 27;18(1):e20534. doi: 10.1002/tpg2.20534 (PMC11729983; doi:10.1002/tpg2.20534)
Supplement: Supplementary file 10 — Figure S10 Synteny map of I. seguinii pseudochromosomes compared to contigs of other species reveals a close relationship, with significant portions of the chromosomes conserved between the species. [file TPG2-18-e20534-s012.docx]

**
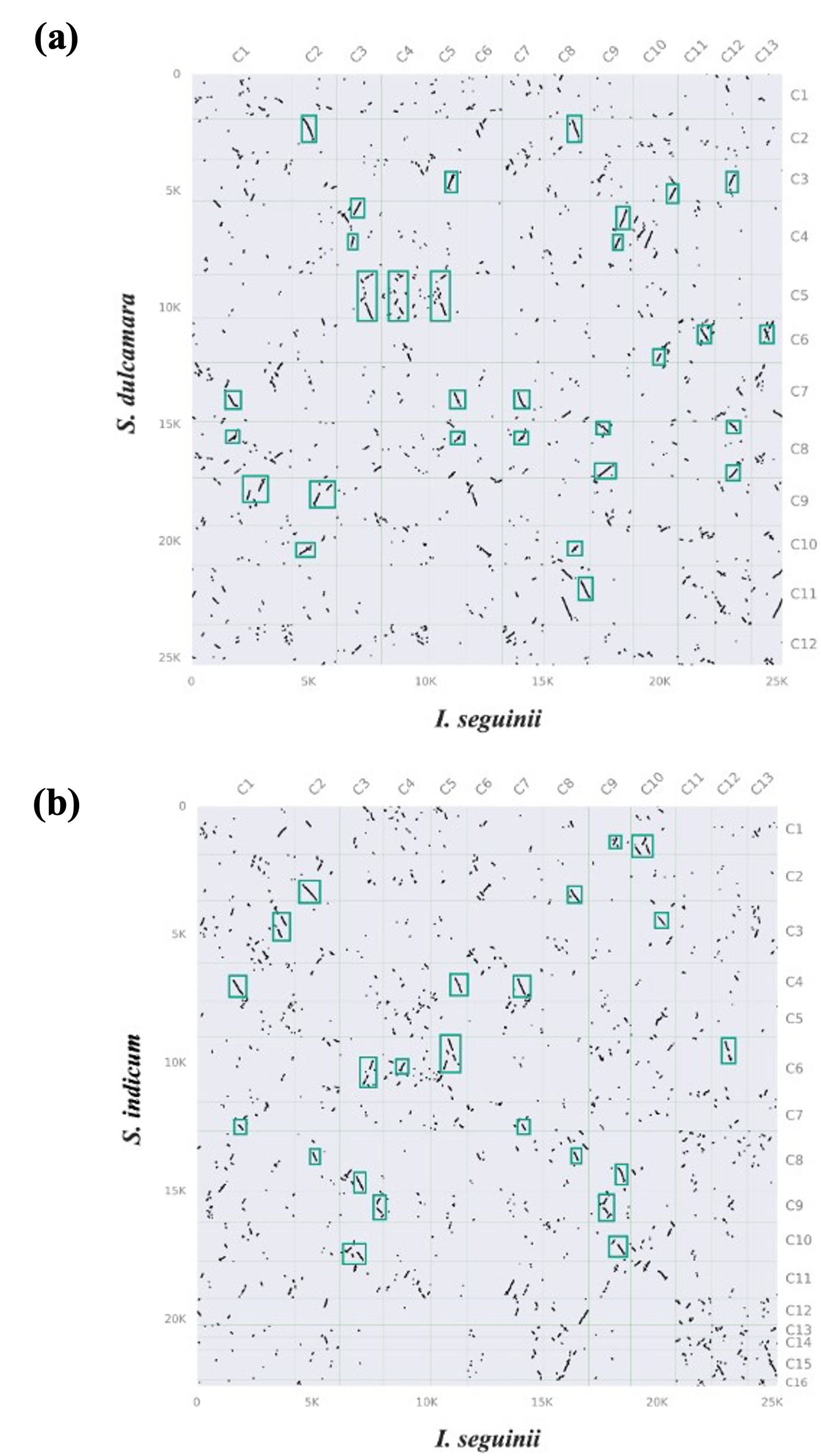
Figure S10 Synteny map of *I. seguinii* pseudochromosomes compared to contigs of other species reveals a close relationship, with significant portions of the chromosomes conserved between the species**. (a) *I. seguinii* & *S. dulcamara*; (b) *I. seguinii* & *S. indicum.*
