# Supplementary material for: Chromosome‐level genome assembly of Iodes seguinii and its metabonomic implications for rheumatoid arthritis treatment
Source: Plant Genome. 2024 Nov 27;18(1):e20534. doi: 10.1002/tpg2.20534 (PMC11729983; doi:10.1002/tpg2.20534)
Supplement: Supplementary file 12 — Figure S12 Comprehensive Analysis of DMs and FCM Clustering. [file TPG2-18-e20534-s009.docx]

**Figure S12 Comprehensive Analysis of DMs and FCM Clustering. (a)** Bar chart represents the number of DMs identified in three comparison groups: leaf vs. stem, root vs. leaf, and root vs. stem. **(b)** Venn diagram illustrates the overlap of DMs among the three comparison groups: leaf vs. stem, root vs. leaf, and root vs. stem, highlighting shared and unique metabolites. **(c)** FCM algorithm analysis of DMs, showcasing the clustering and distribution patterns of the metabolites in the comparison groups.
