# Supplementary material for: Chromosome‐level genome assembly of Iodes seguinii and its metabonomic implications for rheumatoid arthritis treatment
Source: Plant Genome. 2024 Nov 27;18(1):e20534. doi: 10.1002/tpg2.20534 (PMC11729983; doi:10.1002/tpg2.20534)
Supplement: Supplementary file 14 — Table S2 Detailed results from the BOLD for accurate species identification using DNA barcoding. [file TPG2-18-e20534-s006.docx]

## Table S2 Detailed results from the BOLD for accurate species identification using DNA barcoding. IS, *Iodes seguinii*.

| **Sample #** | **Sequence ID** | **Best match in BOLD Systems** | **Score** | **Similarity** |
| --- | --- | --- | --- | --- |
| IS1 | *rbc*L | *Iodes* | 546 | 99.46 |
|  | *mat*K-1 | *Iodes seguinii* | 589 | 100 |
|  | *mat*K-2 | *Iodes seguinii* | 700 | 100 |
|  | *mat*K-3 | *Iodes seguinii* | 794 | 100 |
|  | *mat*K-4 | *Iodes seguinii* | 713 | 100 |
| IS2 | *rbc*L | *Iodes* | 546 | 99.46 |
|  | *mat*K-1 | *Iodes seguinii* | 589 | 100 |
|  | *mat*K-2 | *Iodes seguinii* | 701 | 100 |
|  | *mat*K-3 | *Iodes seguinii* | 786 | 100 |
|  | *mat*K-4 | *Iodes seguinii* | 716 | 100 |
| IS3 | *rbc*L | *Mappianthus iodoides* | 547 | 98.24 |
|  | *mat*K-1 | *Iodes seguinii* | 590 | 100 |
|  | *mat*K-2 | *Iodes seguinii* | 701 | 100 |
|  | *mat*K-3 | *Iodes seguinii* | 802 | 100 |
|  | *mat*K-4 | *Iodes seguinii* | 713 | 100 |
