# Supplementary material for: Chromosome‐level genome assembly of Iodes seguinii and its metabonomic implications for rheumatoid arthritis treatment
Source: Plant Genome. 2024 Nov 27;18(1):e20534. doi: 10.1002/tpg2.20534 (PMC11729983; doi:10.1002/tpg2.20534)
Supplement: Supplementary file 15 — Table S3 Results of sequence alignment for psbA‐trnH against NT database. [file TPG2-18-e20534-s001.docx]

## Table S3 Results of sequence alignment for *psbA-trnH* against NT database. IS, *Iodes seguinii*

| **Sample** | **Sequence ID** | **Best match in NCBI NT** | **Total Score** | **Identity (%)** |
| --- | --- | --- | --- | --- |
| IS1 | *psbA*-*trnH* | *Iodes cirrhosa* chloroplast | 2×10^-96^ | 82.96 |
| IS2 | *psbA*-*trnH* | *Iodes cirrhosa* chloroplast | 7×10^-97^ | 83.00 |
| IS3 | *psbA*-*trnH* | *Iodes cirrhosa* chloroplast | 7×10^-97^ | 83.00 |
