# Supplementary material for: Chromosome‐level genome assembly of Iodes seguinii and its metabonomic implications for rheumatoid arthritis treatment
Source: Plant Genome. 2024 Nov 27;18(1):e20534. doi: 10.1002/tpg2.20534 (PMC11729983; doi:10.1002/tpg2.20534)
Supplement: Supplementary file 17 — Table S5 Length statistics of different types of repeats in the nuclear genome of I. seguinii [file TPG2-18-e20534-s004.docx]

## Table S5 Length statistics of different types of repeats in the nuclear genome of *I. seguinii*

| **Element type** | **Number of elements** | **Length occupied (bp)** | **% of Genome** |
| --- | --- | --- | --- |
| Retroelements | 71,719 | 49,654,039 | 18.15 |
| SINEs | 3,370 | 455,126 | 0.17 |
| LINEs | 16,114 | 10,654,015 | 3.89 |
| LTR elements | 52,235 | 38,544,898 | 14.09 |
| DNA transposons | 22,573 | 11,398,308 | 4.17 |
| Rolling-circles | 525 | 259,728 | 0.09 |
| Unclassified | 144,150 | 48,078,002 | 17.57 |
| Total interspersed repeats | - | 109,130,349 | 39.89 |
| Small RNA | 1,380 | 148,296 | 0.05 |
| Satellites | 168 | 28,330 | 0.01 |
| Simple repeats | 109,130 | 4,788,122 | 1.75 |
| Low complexity | 19,319 | 920,518 | 0.34 |
| Total Repeats identified | - | 115,275,343 | 42.14 |
