# Supplementary material for: Chromosome‐level genome assembly of Iodes seguinii and its metabonomic implications for rheumatoid arthritis treatment
Source: Plant Genome. 2024 Nov 27;18(1):e20534. doi: 10.1002/tpg2.20534 (PMC11729983; doi:10.1002/tpg2.20534)
Supplement: Supplementary file 19 — Table S7 Genome annotation evaluation using BUSCO analysis [file TPG2-18-e20534-s015.docx]

**Table S7 Genome annotation evaluation using BUSCO analysis**

| **Types** | **Number** | **Percentage (%)** |
| --- | --- | --- |
| Complete BUSCOs (C) | 1,513 | 93.74 |
| Complete and single-copy BUSCOs (S) | 1,493 | 92.50 |
| Complete and duplicated BUSCOs (D) | 20 | 1.24 |
| Fragmented BUSCOs (F) | 60 | 3.72 |
| Missing BUSCOs (M) | 41 | 2.54 |
| Total BUSCO groups searched | 1,614 | 100.00 |
