# Supplementary material for: Chromosome‐level genome assembly of Iodes seguinii and its metabonomic implications for rheumatoid arthritis treatment
Source: Plant Genome. 2024 Nov 27;18(1):e20534. doi: 10.1002/tpg2.20534 (PMC11729983; doi:10.1002/tpg2.20534)
Supplement: Supplementary file 22 — Table S10 Molecular docking results of the main components in I. seguinii with the hub targets [file TPG2-18-e20534-s005.docx]

**Table S10 Molecular docking results of the main components in *I*. *seguinii* with the hub targets**

| **Compound** | **Molecular formular** | ***AKT1* affinity score** | ***TLR4* affinity score** | ***EGFR* affinity score** | ***TNF* affinity score** | ***TP53* affinity score** | ***NFKB1* affinity score** | ***JAK2* affinity score** | ***BCL2* affinity score** | ***MAPK1* affinity score** | ***SYK* affinity score** |
| --- | --- | --- | --- | --- | --- | --- | --- | --- | --- | --- | --- |
| Targretin | C_20_H_20_O_7_ | -5.6 | -6.7 | -8.7 | -5.7 | -6.8 | -6.2 | -8.1 | -6.1 | -6.6 | -7.1 |
| Apigenin | C_15_H_10_O_5_ | -5.6 | -7.4 | -9.3 | -6.7 | -7.5 | -6.9 | -9.1 | -6.8 | -7.2 | -8.0 |
| Luteolin | C_15_H_10_O_6_ | -6.3 | -7.5 | -9.6 | -6.8 | -7.7 | -7.5 | -9.1 | -7.0 | -7.6 | -8.3 |
| Jaceosidin | C_17_H_14_O_7_ | -5.9 | -7.3 | -9.6 | -6.4 | -7.5 | -7.0 | -8.6 | -6.9 | -7.2 | -7.9 |
| 6-Hydroxyapigenin | C_15_H_10_O_6_ | -6.5 | -7.4 | -9.6 | -6.4 | -7.7 | -7.1 | -9.3 | -6.9 | -7.6 | -8.3 |
| Kaempferol | C_15_H_10_O_6_ | -6.1 | -7.5 | -9.4 | -6.5 | -7.6 | -6.9 | -9.0 | -6.6 | -7.5 | -7.9 |
| Butein | C_15_H_12_O_5_ | -6.2 | -7.1 | -9.2 | -6.2 | -7.2 | -6.3 | -9.2 | -7.0 | -7.2 | -7.6 |
| Morin | C_15_H_10_O_7_ | -6.3 | -7.1 | -9.4 | -6.6 | -7.6 | -7.1 | -8.9 | -6.9 | -7.3 | -7.9 |
| Isorhamnetin | C_16_H_12_O_7_ | -5.9 | -7.7 | -9.4 | -6.7 | -7.4 | -7.0 | -8.8 | -6.5 | -7.4 | -8.0 |
| Quercetin | C_15_H_10_O_7_ | -6.1 | -8.0 | -9.5 | -6.8 | -7.8 | -6.9 | -9.0 | -6.8 | -7.6 | -8.3 |
| Fisetin | C_15_H_10_O_6_ | -6.5 | -7.9 | -9.6 | -6.9 | -7.6 | -6.8 | -9.8 | -7.1 | -7.8 | -7.8 |

* The numbers in the table are in units of kcal/mol.
